# RAF1 scaffold integrity shapes chemogenetic degradation outcomes in KRAS-driven lung cancer models

**DOI:** 10.26508/lsa.202603839

**Published:** 2026-09-22

**Authors:** Laura de-la-Puente-Ovejero, Ana Domostegui, Inés María García-Pérez, Gonzalo Aizpurua, Lucía Lomba-Riego, Pilar Ximenez-Embún, Cristina Mayor-Ruiz, Mariano Barbacid, Sara García-Alonso

**Affiliations:** 1 Experimental Oncology Group, Tumor Biology Programme, Centro Nacional de Investigaciones Oncológicas (CNIO) , Madrid, Spain; 2 Centro de Investigación Biomédica en Red de Cáncer (CIBERONC), Instituto de Salud Carlos III, Madrid, Spain; 3 Institute for Research in Biomedicine (IRB Barcelona), Barcelona, Spain; 4 Proteomics Unit, Biotechnology Program, Centro Nacional de Investigaciones Oncológicas (CNIO) , Madrid, Spain

## Abstract

Scaffold integrity is essential for the activity of proteins that function through protein–protein interactions rather than catalytic output. RAF1 exemplifies this duality: although it is a bona fide kinase and a core component of the MAPK cascade, its tumor-promoting role is largely kinase-independent, relying instead on scaffold-mediated suppression of apoptosis. Genetic *Raf1* ablation in KRAS-driven lung adenocarcinoma mouse models induces tumor regression without systemic toxicity, making it an attractive candidate for targeted protein degradation. Chemogenetic systems like the dTAG platform are widely used for preclinical target validation. Here, we generated a dTAG-RAF1 mouse model and showed that pharmacological degradation is efficient and systemically well tolerated but fails to reproduce the tumor regression observed upon genetic *Raf1* ablation. Mechanistically, the N-terminal FKBP12^F36V^ tag (dTAG) perturbs the RAF1 interactome, including scaffold associations with apoptotic regulators, thereby blunting the phenotypic consequences of its degradation. These results establish scaffold integrity as a determinant of chemogenetic system fidelity and argue that degradation tools must be validated at the functional level, not only for target elimination, before assessing their therapeutic relevance.

## Abstract (precis)

This study shows that an N-terminal degradation tag can alter RAF1 function before protein removal, highlighting the need to validate tagged proteins in disease models.

## Introduction

Mutations in the RAS gene family occur in ∼30% of all human cancers, with KRAS being the most frequently altered isoform (Prior et al, 2012). These mutations are particularly prevalent in pancreatic, colorectal, and lung adenocarcinomas (LUADs) and are strongly associated with poor prognosis (Kodaz, 2017). Despite the central role of KRAS in tumorigenesis, it was long considered “undruggable” (Moore & Malek, 2021). The discovery of a small, druggable pocket in the KRAS^G12C^ isoform (Ostrem et al, 2013) enabled the development of selective inhibitors such as sotorasib and adagrasib, both of which have received FDA approval (Skoulidis et al, 2021; Jänne et al, 2022; Yaeger et al, 2023). However, clinical responses remain limited by modest efficacy in some settings and by the emergence of adaptive and acquired resistance mechanisms (Arbour et al, 2023; Strickler et al, 2023). As a result, research has expanded toward pan-RAS inhibitors, combination therapies, and targeting of downstream signaling nodes, particularly the RAF/MEK/ERK cascade, a critical axis in RAS-driven malignancies (Barrambana et al, 2025).

Among RAF kinases, RAF1 (also known as CRAF) has emerged as a relevant downstream effector and potential therapeutic target in KRAS-driven LUADs. Conditional deletion of *Raf1* in genetically engineered mouse models (GEMMs) induces tumor regression without major toxicity in normal tissues, indicating that RAF1 is essential for tumor maintenance but dispensable for adult tissue homeostasis (Sanclemente et al, 2018). Moreover, *Raf1* ablation alone is sufficient to promote tumor regression in this setting, with co-targeting of related kinases or receptor tyrosine kinases providing no additional benefit (de-la-Puente-Ovejero et al, 2026). This contrasts with MEK or ERK inhibition, which, although effective in preclinical tumor models, results in systemic toxicity due to the essential roles of these kinases in normal physiology (Drosten & Barbacid, 2020). Interestingly, the tumor-suppressive effects of RAF1 do not arise from the loss of canonical MAPK signaling, but rather from the activation of pro-apoptotic programs normally repressed by RAF1 kinase–independent functions (Sanclemente et al, 2021). Here, we refer to these non-catalytic, protein interaction–mediated activities as RAF1 scaffold functions. In this context, scaffold integrity refers to the ability of RAF1 to preserve the interaction surfaces and protein associations required for its non-catalytic functions.

These features make RAF1 a challenging target for conventional pharmacological approaches. Unlike BRAF, which has a relatively accessible ATP-binding pocket suitable for type I/II kinase inhibitors (Cook & Cook, 2021), the RAF1 kinase domain is conformationally shielded by the HSP90–CDC37 chaperone complex (García-Alonso et al, 2022), limiting accessibility to typical small molecules. Furthermore, pharmacological inhibition of RAF1 catalytic activity is unlikely to fully reproduce the biological consequences of RAF1 loss, given that its tumor-promoting effects in KRAS-driven LUAD are largely non-catalytic. Together, these observations support approaches aimed at eliminating RAF1 protein rather than inhibiting its kinase activity.

Targeted protein degradation (TPD) is a pharmacological strategy that enables the modulation of protein abundance. “Degraders” such as proteolysis targeting chimeras (PROTACs), molecular glues, and other bifunctional molecules exploit the ubiquitin–proteasome system to induce selective elimination of proteins of interest (POIs) (Békés et al, 2022). By recruiting an E3 ubiquitin ligase to a target protein, degraders promote ubiquitination and subsequent proteasomal degradation of the POI. Unlike occupancy-driven inhibitors, TPD has the potential to suppress both catalytic and non-catalytic protein functions by removing the target protein itself (Paiva & Crews, 2019). This makes degradation-based approaches particularly attractive for proteins like RAF1, whose oncogenic functions extend beyond enzymatic activity.

Nevertheless, applying TPD to scaffold proteins presents specific challenges. First, degrader molecules must engage structurally accessible surfaces on the POI, which can be difficult when the target is embedded in large regulatory complexes, such as RAF1 within the RAF1–HSP90–CDC37 (RHC) complex (García-Alonso et al, 2022). Second, efficient protein elimination does not necessarily phenocopy genetic ablation of the corresponding gene, and the functional consequences of these two interventions must therefore be evaluated independently. Third, some proteins, such as RAF1, currently lack high-affinity selective ligands; in those cases, chemogenetic degradation systems are often used for initial therapeutic validation (Mayor-Ruiz & Winter, 2019). Widely used systems such as the degradation TAG (dTAG) system (Nabet et al, 2018) enable the rapid and selective elimination of otherwise undruggable proteins. However, the required N- or C-terminal fusion of chemogenetic tags to the POI can sometimes disturb native conformation, subcellular localization, or interaction networks, thereby complicating biological interpretation (Taylor et al, 2025).

This possibility is not unique to chemogenetic systems, as the ability of N- or C-terminal fusion tags to perturb protein function has long been recognized. In the context of induced protein degradation, however, such preexisting alterations are particularly relevant because they can mislead comparisons between degradation of the tagged protein and genetic ablation of the endogenous target. Consistent with this concern, recent studies have shown that chemical degradation of these chimeric proteins may yield weaker or divergent phenotypes compared with genetic ablation (Barsoum et al, 2023; Yenerall et al, 2023). Therefore, it is essential to validate degradation tools not only at the level of target elimination but also at the level of functional equivalence to the endogenous protein.

In this study, we used dTAG-RAF1 as a model to evaluate both the feasibility of inducible protein degradation in vivo and the functional fidelity of chemogenetic tagging applied to an oncogenic protein. We generated a conditional KRAS-driven LUAD mouse model in which endogenous *Raf1* was replaced by a chemically degradable 2xHA-FKBP12^F36V^–tagged (dTAG) variant. Using this system, we assessed systemic tolerability, tumor response, MAPK signaling, apoptosis, and RAF1-associated protein interactions after degradation with the VHL-recruiting PROTAC dTAG^V^-1m. Unexpectedly, although dTAG-RAF1 was efficiently degraded and systemic RAF1 elimination was well tolerated, the tagged protein was not fully functionally equivalent to endogenous RAF1. The N-terminal tag selectively impaired apoptosis-associated interactions while preserving MAPK-associated signaling, thereby limiting the ability of the model to reproduce the effects of genetic *Raf1* ablation. As an exploratory resource, we also annotated reported RAF1 ubiquitination sites onto the RHC complex to identify solvent-exposed lysines within the native chaperone-bound state. Collectively, our findings emphasize that chemogenetic degradation systems for scaffold proteins require validation beyond degradation efficiency, particularly when the relevant biological activity depends on protein–protein interactions rather than catalytic output.

## Results

### N-terminal FKBP12^F36V^-RAF1 preserves MAPK signaling but alters KRAS-driven tumor progression

**Figure S1.**
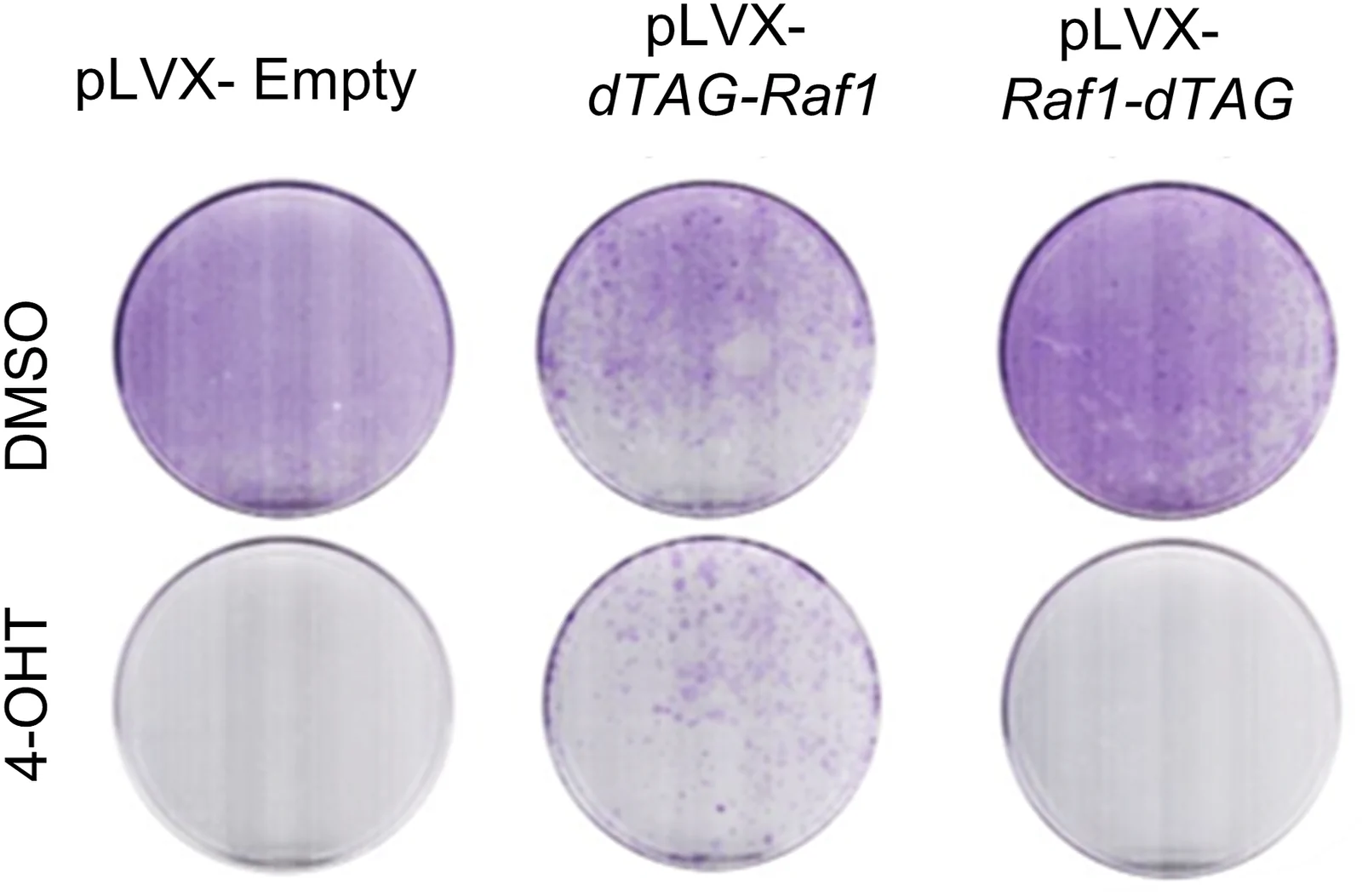
N-terminal dTAG fusion partially restores RAF1 function in RAFless cells. Colony formation assays in RAFless cells transfected with empty vector (pLVX-Empty), dTAG-RAF1 (pLVX-dTAG-*Raf1*), or RAF1-dTAG (pLVX-*Raf1*-dTAG). Cells were treated with DMSO (control) or 4-OHT to eliminate endogenous RAF expression.

To establish a chemogenetic system suitable for inducible RAF1 degradation, we first evaluated whether fusion of dTAG to RAF1 interfered with RAF1 function. N- and C-terminal tagged RAF1 constructs were tested in RAF-deficient (RAFless: *Araf*^L/Y^*; Braf*^L/L^*; Raf1*^L/L^) cells lacking endogenous ARAF, BRAF, and RAF1 expression after 4-hydroxytamoxifen (4-OHT) treatment. Whereas the N-terminal dTAG-RAF1 fusion partially restored colony formation, the C-terminal tag insertion failed to rescue proliferation (Fig S1), indicating that C-terminal tagging compromises RAF1 functionality. Based on these observations, the N-terminal configuration was selected for subsequent studies.

**Figure 1.**
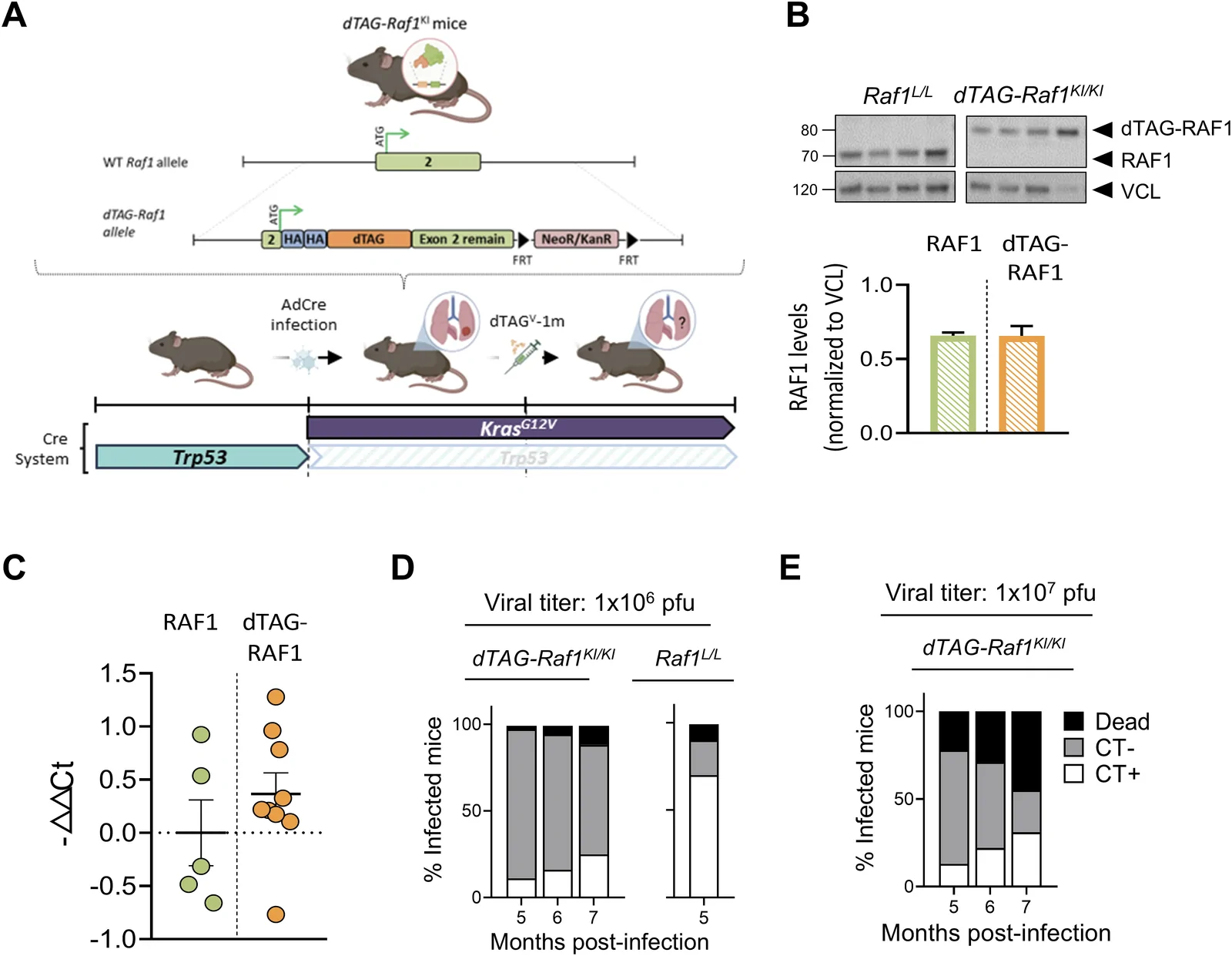
Expression of dTAG-RAF1 delays KRAS-driven lung tumor development. **(A)** Schematic of the *dTAG-Raf1*^KI^ mouse model and experimental design. The endogenous *Raf1* translation initiation codon (ATG) is located in exon 2. This exon (green) was modified to include a double HA tag (blue) followed by the FKBP12^F36V^ sequence (orange), with a NeoR/KanR selection cassette (pink) flanked by FRT sites inserted downstream. *dTAG-Raf1*^KI/KI^ mice were infected intranasally with AdCre to induce *Kras*^G12V^ expression and *Trp53* deletion in lung epithelial cells, generating LUAD. Tumor-bearing mice were subsequently treated with dTAG^V^-1m to achieve pharmacological RAF1 elimination at the protein level. Solid-colored boxes indicate expressed alleles; striped boxes represent ablated genes. **(B)** Representative Western blots showing RAF1 and dTAG-RAF1 protein levels in lung tissue from n = 4 mice per genotype. RAF1 and dTAG-RAF1 signals were quantified using ImageJ software and normalized to the corresponding VCL signal present on the same membrane. **(C)** qRT-PCR quantification of *Raf1* mRNA in lung tissue from n = 5 (*Raf1*^L/L^) and n = 8 (*dTAG-Raf1*^KI/KI^) mice. Ct values were normalized to the housekeeping control (*Rn18s*), and the mean ΔCt of the *Raf1*^L/L^ group was used as the reference. Data are presented as −ΔΔCt, corresponding to log_2_-transformed relative expression. Data in B and C are presented as mean ± SEM of two independent experiments. Statistical comparisons were performed using unpaired *t* test; no significant differences were detected. **(D, E)** Tumor onset in *Kras*^+/G12V^; *Trp53*^−/−^; *dTAG-Raf1*^KI/KI^ and control (*Kras*^+/G12V^; *Trp53*^−/−^; *Raf1*^L/L^) mice after infection with 1 × 10^6^ or 1 × 10^7^ pfu AdCre. Tumor detection by CT scan is shown as a percentage of total infected mice, classified as tumor-free (CT-negative, grey), tumor-bearing (CT-positive, white), or deceased (black) at each time point. Panel D includes 87 dTAG*-Raf1*^KI/KI^ mice and 55 *Raf1*^L/L^ mice from a historical cohort, shown at 5 mo post-infection for reference; panel E includes 45 dTAG-*Raf1*^KI/KI^ mice.

Using homologous recombination, we generated a knock-in mouse model in which the endogenous *Raf1* allele was replaced by a chemically degradable 2xHA-FKBP12^F36V^–tagged version (dTAG-RAF1), enabling pharmacological control of RAF1 abundance (Fig 1A). Expression analysis confirmed that dTAG-RAF1 was produced at levels comparable to endogenous RAF1 at both the protein and mRNA levels (Fig 1B and C).

To determine whether expression of the tagged protein affected KRAS-driven tumorigenesis, *Kras*^+/LSLG12V^; *Trp53*^L/L^; *dTAG-Raf1*^KI/KI^ mice were infected intranasally with AdCre and monitored by computerized tomography (CT) imaging. Kras^G12V^-driven, Trp53-null LUAD models have been routinely used in our laboratory across multiple studies and independently generated cohorts, consistently yielding CT-detectable tumors within approximately 5–6 mo after viral induction (Sanclemente et al, 2018; de-la-Puente-Ovejero et al, 2026). Against this well-established temporal benchmark, only a minority of *Kras*^+/LSLG12V^; *Trp53*^L/L^; *dTAG-Raf1*^KI/KI^ mice showed CT-detectable lesions even after prolonged follow-up (Fig 1D). Increasing the viral titer tenfold did not enhance tumor penetrance and instead increased toxicity without improving tumor burden (Fig 1E). Although the historical *Kras*^+/LSLG12V^; *Trp53*^L/L^; *Raf1*^L/L^ cohort was not generated contemporaneously and potential cohort-related effects cannot be formally excluded, the marked deviation from the tumor-onset kinetics reproducibly observed in this model is consistent with partial impairment of RAF1-dependent tumor-promoting functions in vivo, despite preserved expression levels of the dTAG-RAF1.

**Figure S2.**
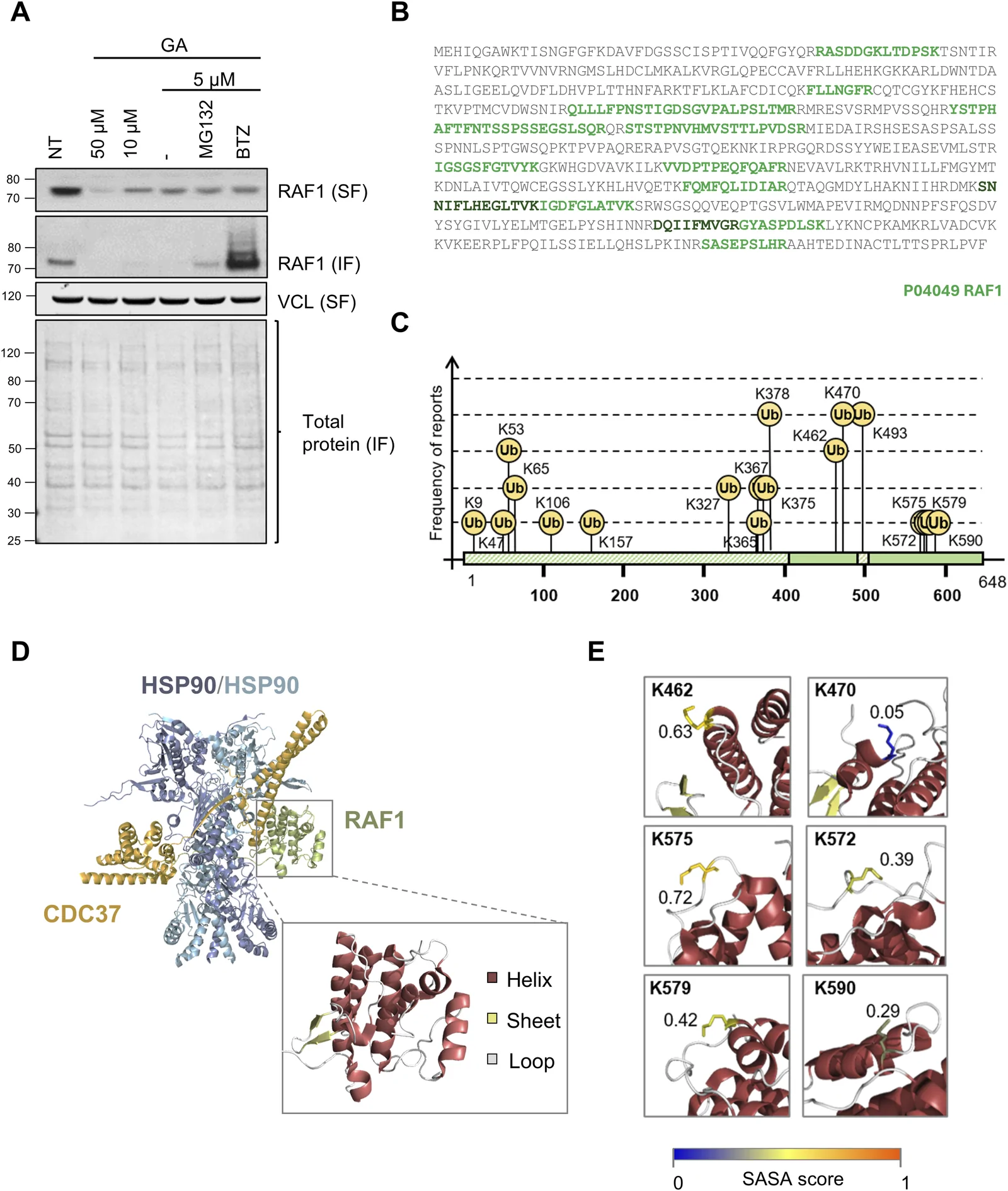
Characterization of the RAF1 ubiquitination landscape and structural mapping of surface-accessible lysines. **(A)** Western blot analysis of RAF1 levels in A549 cells treated with GA alone or in combination with proteasome inhibitors (MG132 or BTZ), both in soluble and insoluble fractions. **(B)** RAF1 (P04049) peptides identified in SDS–PAGE bands analyzed by mass spectrometry are represented in green. Light and dark shades are used to distinguish different but consecutive peptides in the amino acid sequence. **(C)** Summary of high-confidence ubiquitin sites identified from ubiquitinome repositories, prediction tools, and large-scale datasets. **(D)** Mapping of ubiquitinated lysines onto the cryo-EM structure of the RHC complex (PDB: 7Z38), highlighting the secondary structure context of each resolved lysine. **(E)** Close-up views of the six structurally resolved lysines, colored by solvent-accessible surface area (SASA) score as indicated.

As an exploratory resource for future RAF1 degrader development, we also annotated reported RAF1 ubiquitination sites onto available RAF1 structural models. Direct experimental detection of RAF1 ubiquitinated peptides after HSP90 destabilization and proteasome inhibition was technically challenging, likely because of poor recovery from insoluble material (Fig S2A and B). Integration of large-scale ubiquitinome datasets with curated repositories and structural mapping identified K462 and K575 as the most solvent-exposed lysines among the structurally resolved RAF1 ubiquitination sites (Fig S2C and E). These observations do not predict degrader-productive ubiquitination but provide structural annotation of accessible RAF1 surfaces within the native RHC complex.

### Systemic degradation of dTAG-RAF1 is well tolerated in adult mice

We next assessed whether pharmacological elimination of dTAG-RAF1 produced systemic toxicity in vivo. Adult tumor-free *Kras*^+/LSLG12V^; *Trp53*^L/L^; *dTAG-Raf1*^KI/KI^ mice (not infected with AdCre) were treated daily for 30 d with the VHL-recruiting degrader dTAG^V^-1m (Martín et al, 2026) (40 mg/kg, i.p.). In parallel, a second compound, IGP002 (a PROTAC analogue with improved solubility and metabolic stability [Martín et al, 2026]) was also evaluated.

**Figure 2.**
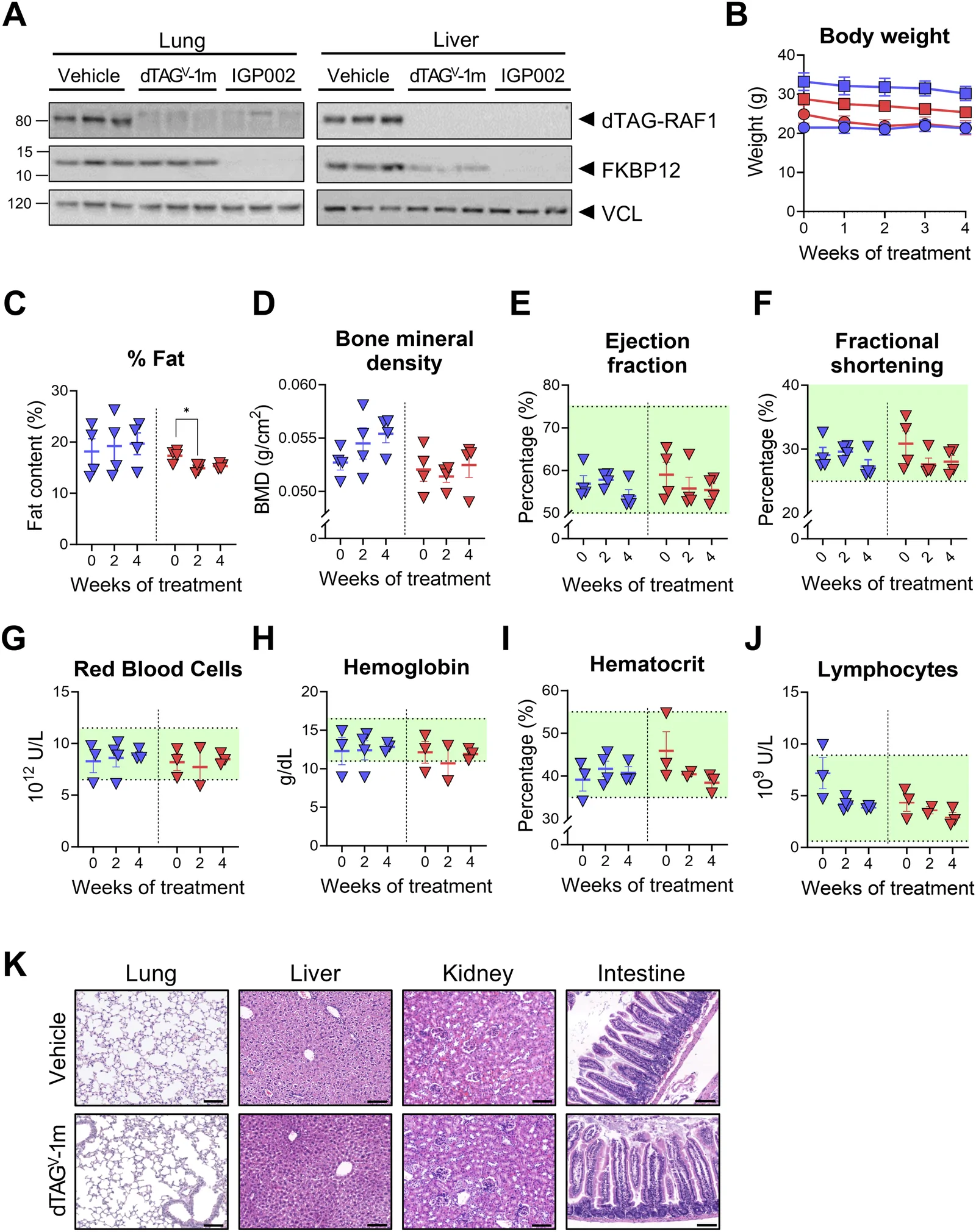
Systemic dTAG^V^-1m–induced degradation of RAF1 is well tolerated in vivo. **(A)** Western blot showing effective degradation of dTAG-RAF1 across major tissues after daily dTAG^V^-1m and IGP002 administration (40 mg/kg) for 30 d. **(B)** Body weight profiles of vehicle- and dTAG^V^-1m–treated *Kras*^+/LSLG12V^; *Trp53*^L/L^; *dTAG-Raf1*^KI/KI^ mice. Males and females are represented by squares and circles, respectively. **(C, D)** Densitometric analysis of body fat percentage and bone mineral density using DEXA scanning. **(E, F)** Echocardiographic assessment of ejection fraction and fractional shortening. **(G, H, I, J)** Hematologic parameters, including RBC count, HGB, HCT, and lymphocyte percentage. Day 0 indicates the baseline measurement obtained before treatment initiation. Data are shown as individual values and mean ± SEM. Vehicle- and dTAG^V^-1m–treated groups are shown in blue and red, respectively. Green shaded areas in panels E, F, G, H, I, J indicate physiological reference ranges. Statistical comparisons were performed using paired *t* test (n = 4 mice per experimental group, comprising 2 males and 2 females). *P < 0.05. **(K)** Representative H&E staining of major organs from vehicle- and dTAG^V^-1m–treated animals. Scale bars: 50 μm.

Western blot analysis confirmed efficient degradation of dTAG-RAF1 in lung and liver after dTAG^V^-1m treatment (Fig 2A). Although IGP002 also induced dTAG-RAF1 degradation, it additionally led to off-target degradation of endogenous FKBP12 and was therefore excluded from further analysis.

Comprehensive physiological characterization revealed no systemic toxicity associated with sustained dTAG-RAF1 degradation. Body weight remained stable in both dTAG^V^-1m– and vehicle-treated groups (Fig 2B). DEXA scans showed no significant changes in body composition or bone mineral density (Fig 2C and D). Echocardiographic assessment showed preserved ejection fraction and fractional shortening across all groups and time points (Fig 2E and F), indicating maintained cardiovascular integrity. Similarly, hematological parameters remained within physiological ranges, with no significant changes in erythroid or lymphoid populations (Fig 2G–J).

Histopathological examination of major organs, including the lung, liver, kidney, and intestine, revealed no detectable pathological alterations or tissue damage after treatment (Fig 2K). No clinical signs of distress or treatment-associated morbidity were observed during the study.

Together, these findings indicate that systemic pharmacological degradation of dTAG-RAF1 is well tolerated in adult mice and support previous genetic evidence that RAF1 loss does not measurably disrupt adult tissue homeostasis under the conditions examined.

### Efficient degradation of dTAG-RAF1 does not recapitulate the tumor regression observed after genetic *Raf1* ablation

To evaluate the consequences of acute RAF1 elimination in established tumors, *Kras*^+/G12V^; *Trp53*^−/−^; *dTAG-Raf1*^KI/KI^ mice bearing CT-confirmed lung tumors (≥5 mm^3^) were treated daily with dTAG^V^-1m (40 mg/kg i.p.). Tumor growth was monitored longitudinally by CT every 15 d.

**Figure 3.**
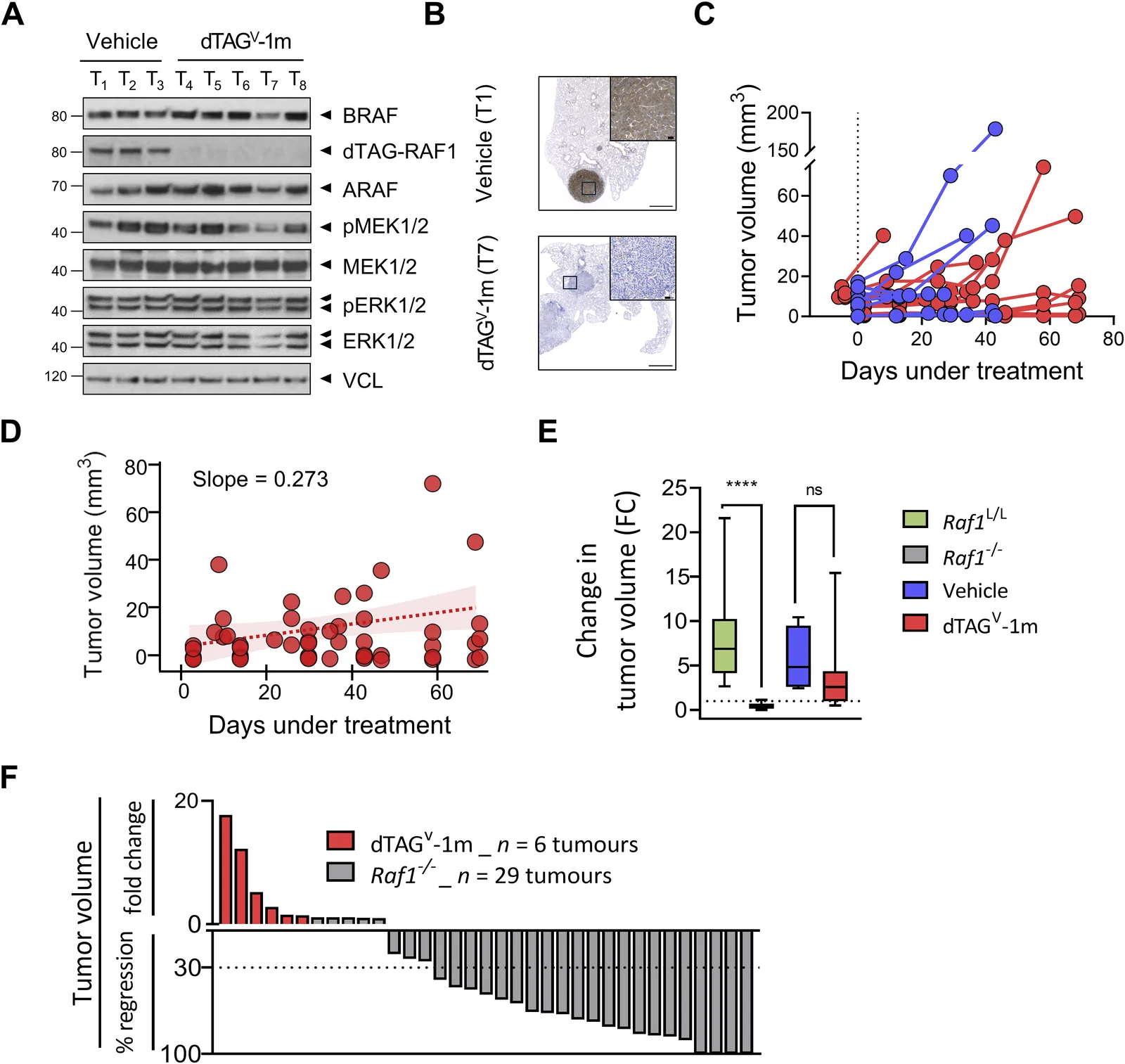
Efficient degradation of dTAG-RAF1 fails to induce tumor regression. **(A)** Western blot validation of dTAG-RAF1 degradation and downstream MAPK pathway in tumor samples. VCL was used as a loading control. **(B)** Representative RAF1 immunostaining of paraffin-embedded lung tumors from vehicle- and dTAG^V^-1m–treated mice. Scale bars: 1 mm (overview), 50 μm (zoom). **(C)** Longitudinal tumor volume measurements by CT imaging of individual tumors from vehicle- (blue; 7 tumors, 6 mice) and dTAG^V^-1m–treated (red; 13 tumors, 7 mice) groups. **(D)** Linear mixed-effects model of tumor growth over time. **(E)** Fold change (FC) in tumor volume for the four cohorts shown, from left to right: historical *Kras*^+/G12V^; *Trp53*^−/−^; *Raf1*^L/L^ control tumors (n = 44 tumors, 12 mice, green); historical *Kras*^+/G12V^; *Trp53*^−/−^; *Raf1*^−/−^ tumors after genetic *Raf1* ablation (n = 40 tumors, 17 mice, grey); vehicle-treated *Kras*^+/G12V^; *Trp53*^−/−^; *dTAG-Raf1*^KI/KI^ tumors (n = 4 tumors, 4 mice, blue); and dTAG^V^-1m–treated *Kras*^+/G12V^; *Trp53*^−/−^; *dTAG-Raf1*^KI/KI^ (n = 9 tumors, 6 mice, red). For each tumor, FC was calculated by dividing its volume at the final CT scan by its volume at treatment initiation; values above 1 indicate tumor growth, whereas values below 1 indicate tumor regression. Only tumors with both baseline and end point measurements were included. n denotes individual tumors. Statistical comparisons were performed between the *Raf1*^L/L^ and *Raf1*^−/−^ cohorts and between the vehicle- and dTAG^V^-1m–treated *dTAG-Raf1*^KI/KI^ cohorts using the Mann–Whitney test; no statistical comparison was performed between historical and contemporaneous cohorts. **(F)** Waterfall plot representing individual tumor volume changes in *Kras*^+/G12V^; *Trp53*^−/−^; dTAG*-Raf1*^KI/KI^ mice treated with dTAG^V^-1m (n = 6, 3 mice, red) and *Kras*^+/G12V^; *Trp53*^−/−^; *Raf1*^−/−^ mice exposed to 4-OHT diet (n = 29, 11 mice, grey) for 2 mo. Comparison with historical *Kras*^+/G12V^; *Trp53*^−/−^; *Raf1*^L/L^ and *Kras*^+/G12V^; *Trp53*^−/−^; *Raf1*^−/−^ cohorts is presented for contextual reference; direct statistical comparison is limited by differences in experimental design, treatment agent, and cohort size. Tumor growth in D was assessed using a linear mixed-effects model. Significance thresholds: ****P < 0.0001.

Western blotting and immunohistochemistry (IHC) confirmed robust degradation of dTAG-RAF1 protein in tumor tissues after treatment (Fig 3A and B). However, despite efficient and sustained protein elimination, tumors failed to regress. Longitudinal CT imaging instead revealed continued tumor growth throughout the treatment period (Fig 3C). Linear mixed-effects modeling confirmed a statistically significant positive slope in tumor volume over time in dTAG^V^-1m–treated mice, indicative of ongoing tumor progression (Fig 3D).

To contextualize these findings, tumor responses in the current vehicle- and dTAG^V^-1m–treated *Kras*^+/G12V^; *Trp53*^−/−^; *dTAG-Raf1*^KI/KI^ cohorts were displayed alongside previously published *Raf1*^L/L^ and *Raf1*^−/−^ cohorts (Sanclemente et al, 2018). Both studies examined CT-positive established Kras^G12V^-driven, Trp53-null LUAD over a 2-mo intervention period. However, they were generated and analyzed independently and, therefore, no statistical comparisons were performed between this genetic ablation model and current-study cohorts. Within these limitations, the data are consistent with the interpretation that pharmacological degradation of dTAG-RAF1 does not reproduce the tumor regression phenotype observed after genetic *Raf1* ablation (Fig 3E and F).

**Figure S3.**
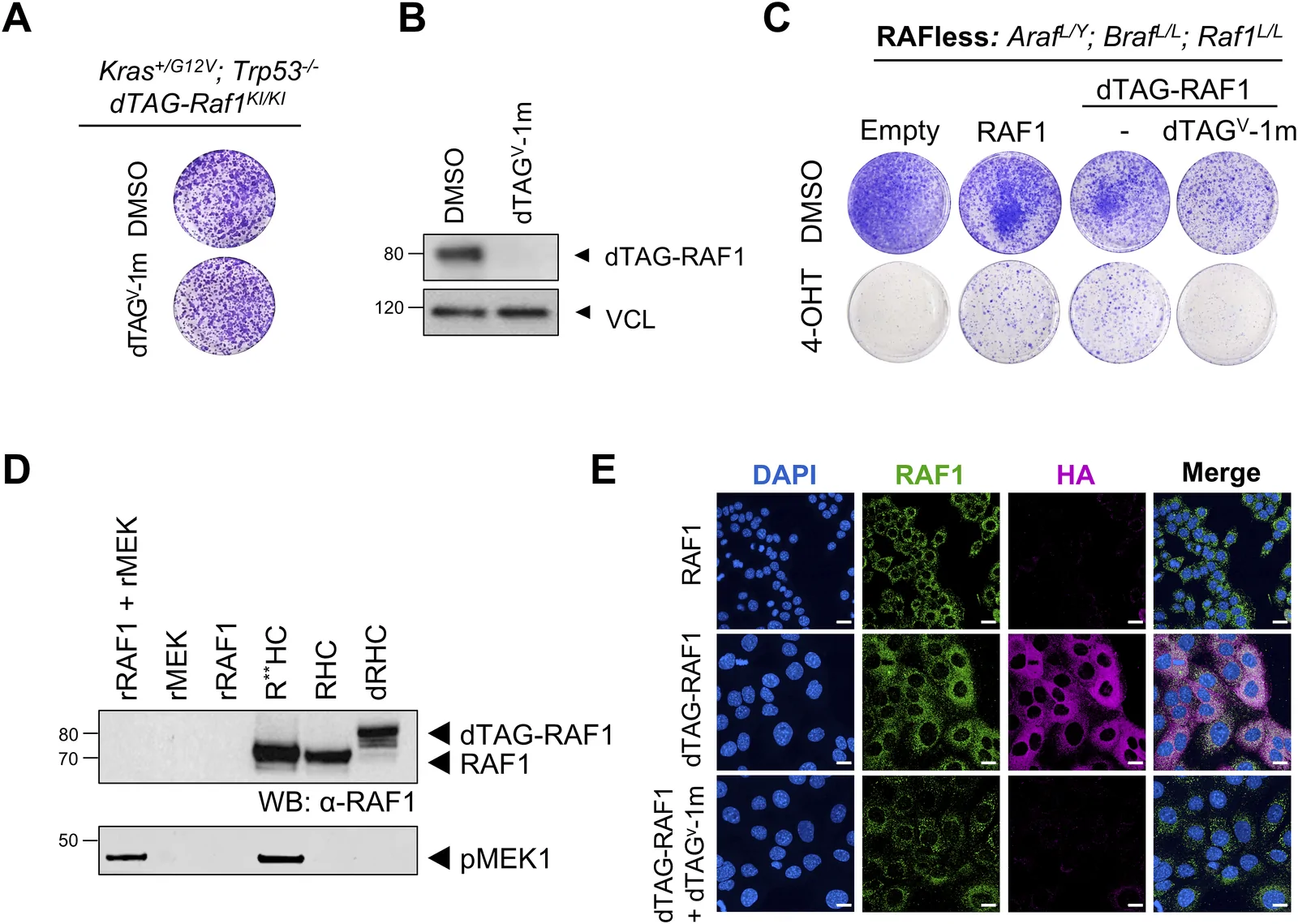
Functional characterization of dTAG-RAF1. **(A)** Representative images of colony formation in LUAD-derived cells treated with DMSO or dTAG^V^-1m for 15 d. **(B)** Western blot analysis confirming chimeric protein degradation in the same cells at end point. **(C)** Colony formation assays in RAFless cells transfected with empty vector (pLVX-Empty), RAF1 (pLVX-*Raf1*) or the chimeric dTAG-RAF1 (pLVX-dTAG-*Raf1*). Cells were exposed to DMSO or 4-OHT to eliminate endogenous RAFs expression. In the case of dTAG-RAF1 treated cells, dTAG^V^-1m was added at 500 nM and refreshed every 2 d. **(D)** In vitro kinase assay using 1 μg recombinant MEK1 (rMEK) as a substrate, added from the same commercial preparation to all reactions. rRAF1 refers to a commercially available, constitutively active recombinant RAF1 (MW = 60 kD; not detected in the RAF1 standard blot because of its lower molecular weight). R** denotes the RAF1^Y340E/Y341E^ constitutively active mutant. dRHC refers to the dTAG–RAF1–HSP90–CDC37 complex. **(E)** Confocal microscopy images illustrating subcellular localization. Scale bar: 20 μm. dTAG^V^-1m: 0.3 μM for 24 h. Images were acquired using a 40× objective.

To complement these in vivo findings, primary LUAD cell lines derived from the same GEMM were established and treated in vitro with dTAG^V^-1m. Consistent with the in vivo response, dTAG-RAF1 degradation did not impair the proliferative capacity of these cells (Fig S3A and B), in contrast to previous observations using WT RAF1-targeting shRNAs, which robustly inhibited colony formation (Sanclemente et al, 2018).

Collectively, these results demonstrate that efficient degradation of dTAG-RAF1 does not phenocopy the antitumor effects previously observed after genetic *Raf1* deletion.

### dTAG-RAF1 preserves MAPK signaling but selectively disrupts scaffold-associated interactions

To investigate why dTAG^V^-1m–mediated RAF1 degradation failed to induce tumor regression, we analyzed whether the dTAG protein retained the signaling and scaffold properties associated with endogenous RAF1.

In RAFless cells, both WT RAF1 and dTAG-RAF1 partially restored colony formation after elimination of endogenous RAF proteins (Fig S3C). In addition, kinase assays showed low detectable phosphorylation of recombinant MEK1 by both WT and dTAG-RAF1 within the HSP90–CDC37 complex context (Fig S3D), consistent with previous observations that RAF1 kinase activity is tightly regulated by chaperone association (Aizpurua et al, 2026 *Preprint*).

Analysis of downstream MAPK signaling showed no major differences between WT and dTAG-RAF1 conditions. ERK phosphorylation remained detectable in tumors before and after dTAG^V^-1m treatment (Fig 3A), and confocal microscopy revealed comparable subcellular localization patterns for WT and dTAG-RAF1 (Fig S3E).

**Figure 4.**
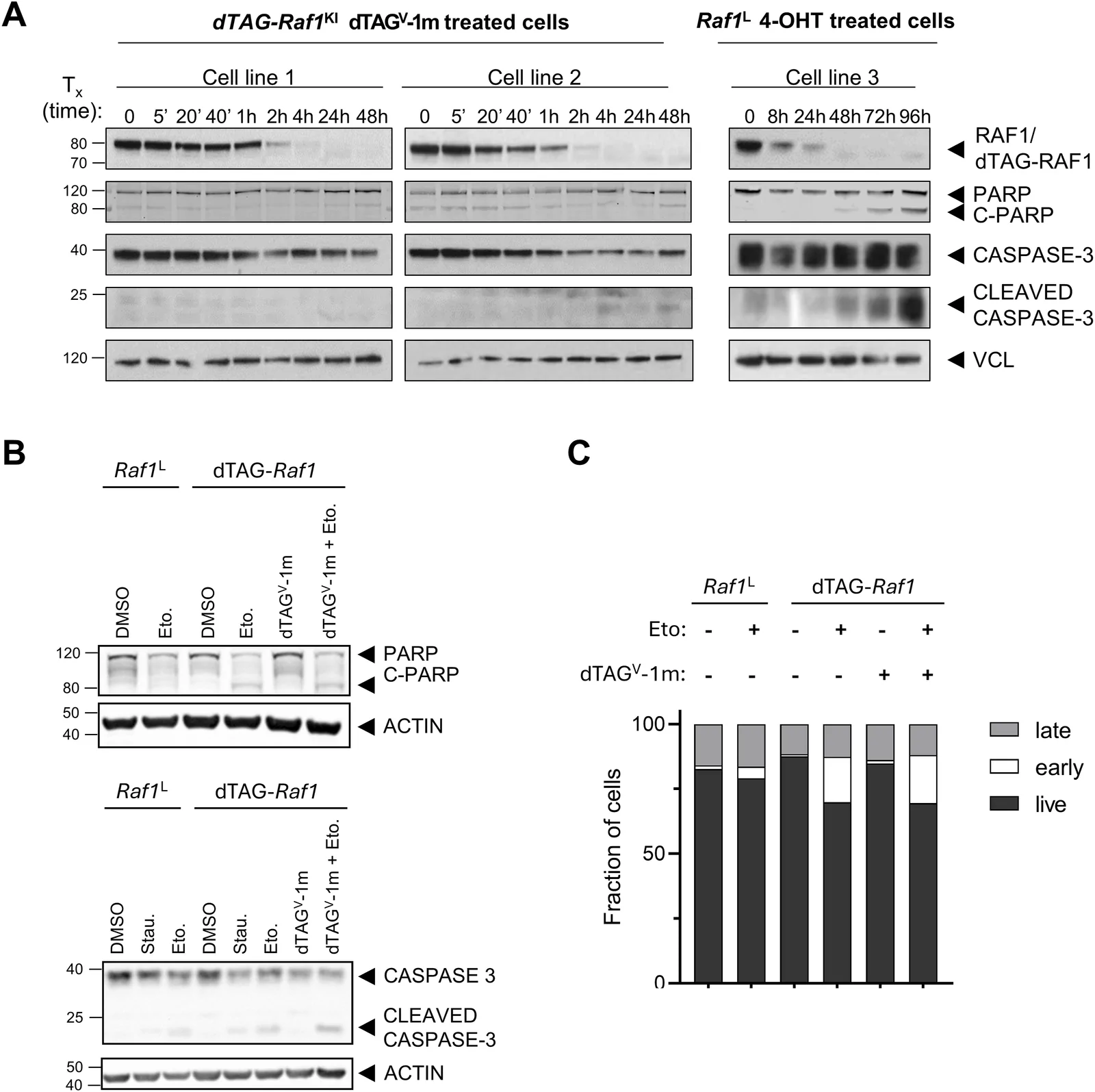
N-terminal dTAG fusion impairs RAF1 scaffold function and apoptotic signaling. **(A)** Western blot analysis of apoptotic markers after RAF1 pharmacological degradation or genetic *Raf1* elimination in independently derived LUAD cell lines. Cells were treated with 300 nM dTAG^V^-1m or 600 nM 4-OHT at the indicated time points, according to the genotype. The different time courses reflect the distinct kinetics of RAF1 elimination. **(B)** Western blot analysis of apoptotic markers in *Raf1*^L^ and *dTAG-Raf1* LUAD-derived cells treated with DMSO, etoposide, dTAG^V^-1m, or the combination of etoposide and dTAG^V^-1m, as indicated. PARP cleavage and caspase-3 cleavage (cl.PARP and cl.CASP3, respectively) were assessed as readouts of apoptotic induction. Actin was used as a loading control. **(C)** Flow cytometric quantification of apoptosis by Annexin V/PI staining across the indicated conditions. Bars represent the fraction of live (Annexin V^−^/PI^−^), early apoptotic (Annexin V^+^/PI^−^), and late apoptotic or necrotic (Annexin V^+^/PI^+^) cells. This panel represents a single exploratory experiment and was not subjected to statistical analysis. All experiments were performed using primary mouse LUAD-derived cell lines of the indicated genotypes. PI, propidium iodide. Source data are available for this figure.

Because RAF1-dependent tumor maintenance has been linked primarily to kinase-independent anti-apoptotic functions, we next examined apoptosis-associated responses. In contrast to the phenotype observed after genetic *Raf1* ablation, degradation of dTAG-RAF1 did not induce detectable accumulation of cleaved caspase-3 (cl.CASP3) (Fig 4A). In *Raf1*^L^ cells, treatment with etoposide or staurosporine produced only limited induction of apoptotic markers, as assessed by cleaved PARP (cl.PARP) and cl.CASP3. In contrast, cells expressing dTAG-RAF1 showed an increase in these markers after the same treatments (Fig 4B). Similar results were obtained when apoptosis was analyzed by flow cytometry (Fig 4C). Together, these data support a model in which *Raf1*^L^ retains anti-apoptotic functions, whereas the N-terminal tag in dTAG-RAF1 compromises scaffold integrity and sensitizes cells to apoptotic stimuli.

**Figure 5.**
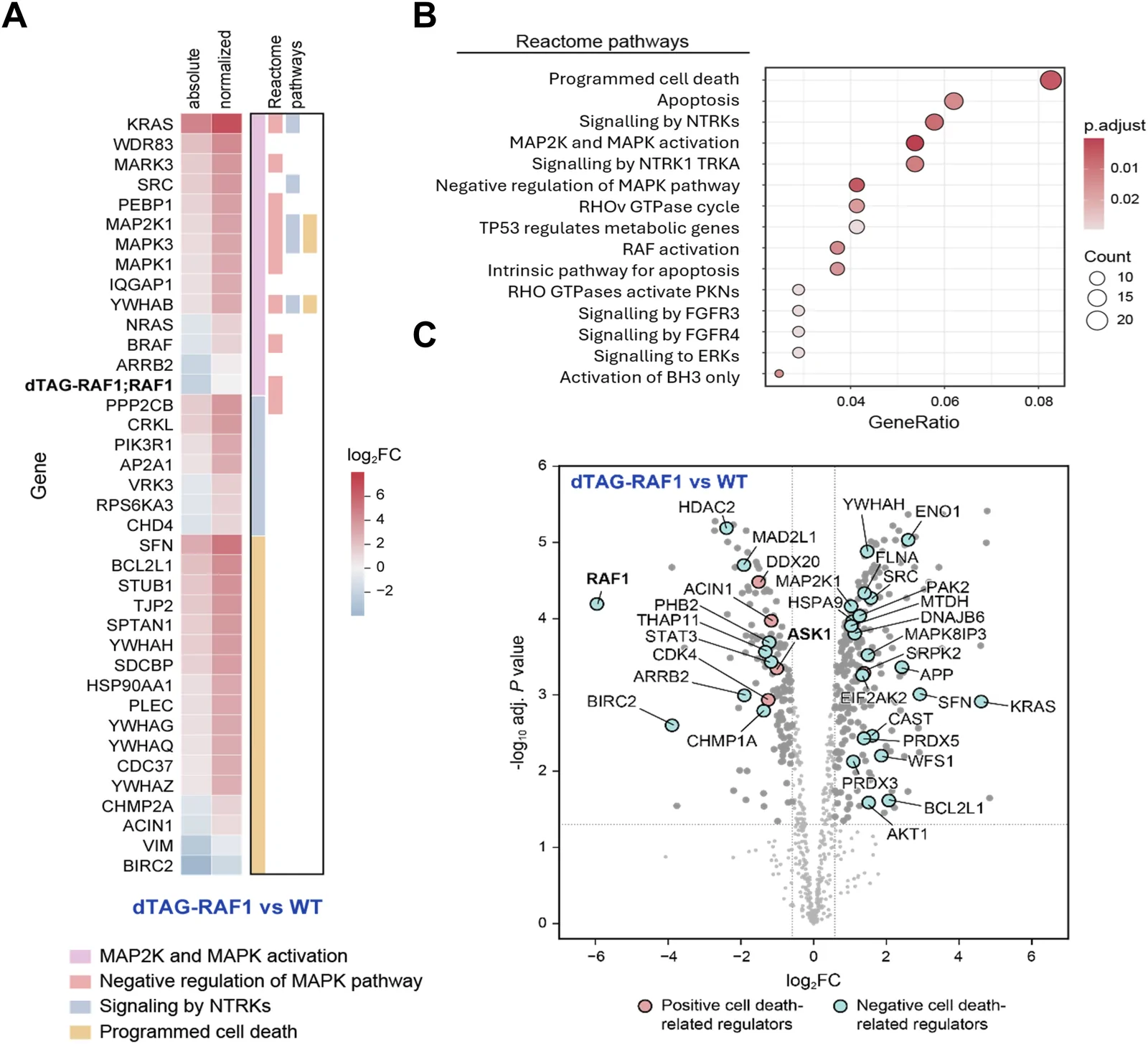
Interactome analysis of dTAG-RAF1 compared with WT RAF1. **(A)** Heatmap showing RAF1-interacting proteins with increased or decreased association with dTAG-RAF1 relative to WT RAF1. Related cellular pathway is also color-coded as indicated. Absolute and RAF1 bait-normalized log_2_ fold changes (FC) are shown. **(B)** Top enriched RAF1-interacting pathways observed in dTAG-RAF1 versus WT RAF1-expressing cells (descriptors from the Reactome Pathway database). **(C)** Volcano plot analysis of positive and negative cell death regulators differentially associated with dTAG-RAF1 relative to WT RAF1. Positive log_2_ FC values indicate increased abundance in the dTAG-RAF1 interactome, whereas negative values indicate increased abundance in the WT RAF1 interactome. Functional annotations describe the reported role of each protein in cell death regulation.

To determine whether the N-terminal dTAG altered the RAF1 interaction landscape, we compared the interactomes of WT RAF1 and dTAG-RAF1 in LUAD-derived cell lines by IP-MS analysis. Although many RAF1-associated proteins were preserved, quantitative comparison identified a subset of interactors with altered abundance in the dTAG-RAF1 pulldown (Fig 5A). Pathway enrichment analysis of these differentially associated proteins revealed a notable overrepresentation of apoptosis and programmed cell death–related pathways (Fig 5B). A more focused pathway analysis using the Reactome database further identified reduced interactions with proteins linked to apoptotic regulation, including ASK1 (Fig 5C), a known RAF1-interacting protein implicated in apoptotic regulation (Chen et al, 2001).

In contrast, no comparable disruption of MAPK-associated interactions was observed, consistent with the maintained ERK phosphorylation detected in vivo. Together, these findings suggest that the N-terminal dTAG does not globally disrupt RAF1 expression or MAPK-associated signaling, but rather alters a subset of RAF1 interactions, including proteins linked to apoptotic regulation.

## Discussion

TPD has emerged as a promising strategy to modulate proteins that remain difficult to inhibit pharmacologically, including multidomain signaling and scaffold proteins. RAF1, a critical effector downstream of KRAS, represents a particularly relevant example in KRAS-driven LUADs, where previous genetic studies demonstrated that *Raf1* ablation promotes robust tumor regression without inducing significant toxicity in healthy tissues (Sanclemente et al, 2018). Notably, this tumor-suppressive effect does not result from inhibition of canonical MAPK signaling but instead relies on the disruption of kinase-independent scaffold functions involved in apoptosis regulation. These findings position RAF1 as a useful model to investigate the biological consequences and therapeutic implications of induced protein degradation in vivo. The contrasting consequences of RAF1 loss in normal tissues and KRAS-driven tumors raise the possibility that oncogenic KRAS rewires effector usage, creating a selective dependence on RAF1 scaffold-mediated suppression of apoptosis rather than simply increasing canonical pathway output. Similar context-dependent engagement or repurposing of RAS effectors has been proposed for PI3K (Gupta et al, 2007; Murillo et al, 2018; Vasjari et al, 2019).

However, targeting RAF1 with small molecules remains challenging. Unlike BRAF, which commonly harbors oncogenic kinase-activating mutations, the RAF1 kinase domain is deeply buried within the HSP90–CDC37 chaperone complex, rendering it sterically inaccessible. Given the absence of selective RAF1 degraders, we employed the dTAG system as a chemogenetic approach to induce acute RAF1 degradation in vivo. Our findings demonstrate that efficient depletion of dTAG-RAF1 does not reproduce the tumor regression phenotype previously observed after genetic *Raf1* deletion. Instead, several lines of evidence indicate that the dTAG-RAF1 protein fails to fully replicate the functions of endogenous RAF1. More broadly, these observations highlight an important limitation of chemogenetic degradation systems when applied to scaffold proteins, whose biological activities rely on dynamic and spatially regulated protein–protein interactions that may be particularly sensitive to steric perturbations introduced by fusion tags. Previous studies using chemogenetic degradation systems have reported divergent phenotypes relative to conventional knockout models for proteins including ASH2L, CDK2, and CDK5 (Barsoum et al, 2023; Yenerall et al, 2023), further highlighting the importance of validating functional equivalence in these degradation platforms. These findings may also have implications for the interpretation of earlier studies relying on N-terminally tagged RAF constructs, particularly those investigating protein interactions or non-catalytic functions, which may warrant re-examination using untagged proteins or alternative tag configurations. However, our results do not imply that all N-terminal RAF tags are functionally disruptive, as their effects are likely to depend on tag identity, linker design, insertion site and experimental context.

As a secondary exploratory analysis, we characterized the RAF1 ubiquitination sites within the native RHC complex. Integration of large-scale ubiquitinome datasets (Udeshi et al, 2020; Hansen et al, 2021; Rivera et al, 2021), curated repositories, and structural accessibility analyses identified K462 and K575 as the most solvent-exposed lysine residues within the resolved RHC structure. However, because degrader-induced ubiquitination depends on ternary complex geometry, E3 ligase identity, linker architecture, and induced proximity, these naturally observed or predicted ubiquitination sites should not be interpreted as direct predictors of degrader-productive ubiquitination. Rather, they provide structural context for accessible RAF1 surfaces that may inform future studies.

Systemic dTAG^V^-1m administration in non-tumor–bearing mice was well tolerated, with no significant toxicities observed across physiological, hematological, and histopathological parameters. These findings are consistent with previous genetic ablation studies and further support the notion that RAF1 is dispensable for normal tissue homeostasis in adult mice. Thus, although the dTAG system should not be considered a clinically optimized degrader strategy, these data indicate that systemic RAF1 elimination can be achieved in vivo without apparent toxicity.

Despite robust and sustained degradation of dTAG-RAF1 in tumor-bearing mice, this intervention did not reproduce the tumor regression phenotype previously observed after genetic *Raf1* deletion. Tumors continued to grow under dTAG^V^-1m treatment, and acute dTAG-RAF1 degradation did not induce detectable apoptotic markers. These observations indicate that efficient elimination of the tagged protein is not, by itself, sufficient to phenocopy genetic *Raf1* loss in this model.

Several converging findings suggest that this discrepancy reflects functional non-equivalence between dTAG-RAF1 and endogenous RAF1. Even before dTAG^V^-1m treatment, mice expressing the chimeric dTAG-RAF1 protein showed delayed tumor onset and reduced tumor penetrance, suggesting that the N-terminal dTAG partially impairs RAF1-dependent tumor-promoting functions. Although ERK phosphorylation and subcellular localization of dTAG-RAF1 were preserved, quantitative interactome profiling identified a subset of apoptosis-related proteins with altered association in the dTAG context, including ASK1 (Chen et al, 2001). Pathway enrichment analysis supported a broader pattern of selective reduction in apoptosis-associated interactors in the dTAG context, whereas associations with MAPK effectors remained preserved. Together, these findings are consistent with a model in which dTAG cassette selectively perturbs RAF1 scaffold-mediated apoptotic regulation without globally disrupting RAF1 expression or canonical MAPK signaling.

**Figure 6.**
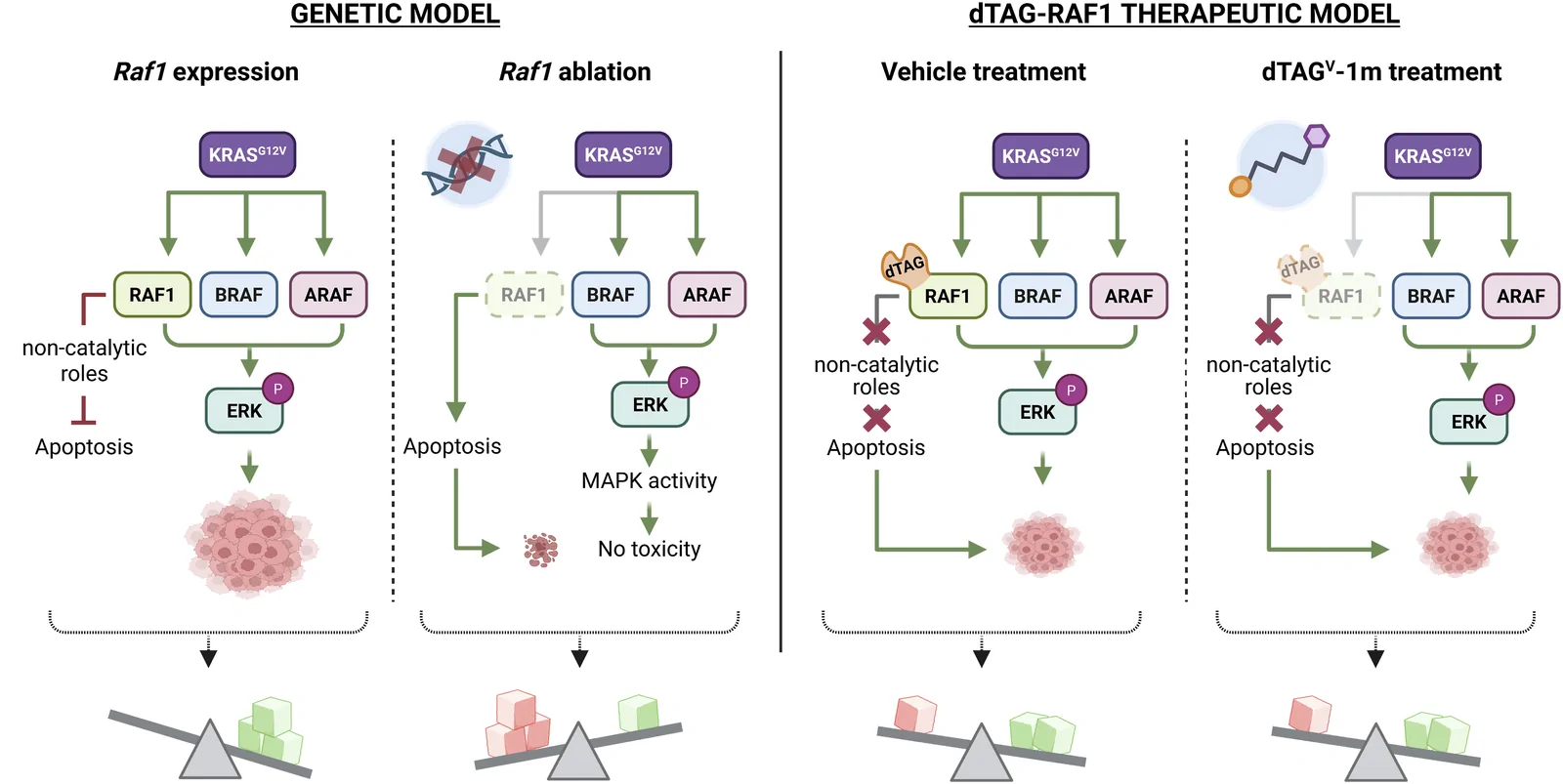
Model for RAF1 function in tumor progression and response to degradation. In *Kras*^G12V^-driven LUAD expressing endogenous RAF1 (*Raf1*^L/L^), canonical MAPK signaling is preserved and RAF1 scaffold functions contribute to suppression of apoptotic signaling, supporting tumor progression. Genetic ablation of RAF1 (*Raf1*^−/−^) disrupts this balance, triggering apoptosis and tumor regression. In tumors expressing the dTAG-RAF1 chimeric protein, MAPK-associated signaling remains intact, but scaffold-mediated apoptotic regulation appears partially impaired, contributing to delayed and reduced tumor progression. Upon dTAG^V^-1m treatment, dTAG-RAF1 is efficiently degraded; however, this does not reproduce the tumor regression observed after genetic *Raf1* ablation. Balance scales illustrate the proposed relationship between proliferative signals (green cubes) and apoptotic regulation (red cubes) in each context.

Based on these findings, we propose a model in which preserved MAPK-associated signaling and altered apoptotic regulation may account for the inability of dTAG-mediated RAF1 degradation to phenocopy genetic *Raf1* ablation (Fig 6). In the genetic model, *Raf1* deletion induces robust tumor regression associated with increased apoptotic signaling, whereas canonical MAPK signaling remains unaltered. In contrast, in the dTAG model, MAPK-associated signaling is preserved, but RAF1 scaffold-mediated apoptotic regulation appears to be partially compromised before degrader treatment. As a result, tumors that emerge in the *dTAG-Raf1*^KI/KI^ background may be less dependent on RAF1-mediated apoptotic suppression, limiting the phenotypic consequences of subsequent dTAG-RAF1 degradation. This model illustrates that degrading a functionally compromised protein does not necessarily replicate the effects of genetic ablation.

Beyond RAF1, these findings are relevant to a broader class of scaffolds and regulatory proteins whose biological functions are not fully captured by catalytic activity. For such proteins, degradation may offer advantages over conventional inhibition, but our results underscore that chemogenetic degradation models must preserve the interaction-dependent functions that define target biology. Fusion of chemogenetic tags, although useful for conditional degradation, can disrupt protein–protein interactions and thereby complicate interpretation of target dependency and degradation phenotypes. Structure-guided allele design may help prioritize alternative tag positions. Although no internal site can be assumed to be functionally neutral, candidate insertion sites could initially be selected within predicted solvent-exposed, low-confidence or intrinsically disordered segments, while avoiding the structured RAS-binding and cysteine-rich domains, the kinase domain, and critical regulatory motifs such as the region surrounding S259. For RAF1, the disordered region separating its N-terminal regulatory modules from the kinase domain could therefore provide candidate positions for empirical screening. However, structural predictions alone cannot establish functional neutrality. Candidate alleles should be assessed at physiological expression levels for protein abundance and localization, kinase activity, relevant protein–protein interactions, and the ability to support RAF1-dependent biological functions before their incorporation into in vivo degradation models. More generally, degrader screening and validation platforms should incorporate interactome profiling and functional readouts, such as apoptosis, differentiation, or tumor regression, in addition to measurements of degradation efficiency or target abundance.

Several limitations of this study should be acknowledged. This work focuses on a single scaffold protein, one N-terminal chemogenetic tag configuration, and one degrader. Therefore, these findings should not be generalized to all degron-based approaches but rather identify a potential source of functional perturbation that should be evaluated on a case-by-case basis. The tumor treatment cohort was small, mainly because of the reduced tumor penetrance observed in dTAG-Raf1^KI/KI^ mice. Comparisons with historical *Raf1*^L/L^ and *Raf1*^−/−^ cohorts are provided for contextual reference only, as differences in experimental design, treatment regimen, and cohort size preclude direct statistical comparison. Finally, because the dTAG system does not recapitulate the pharmacological properties of a bona fide RAF1 degrader, conclusions regarding the therapeutic potential of RAF1 elimination should be interpreted within this context.

In summary, our study shows that systemic RAF1 elimination using a chemogenetic degradation system is well tolerated in vivo, but also reveals that N-terminal FKBP12^F36V^ tag selectively alters RAF1 scaffold-associated functions. Consequently, pharmacological degradation of dTAG-RAF1 does not phenocopy genetic *Raf1* ablation. This work reinforces the concept that, for targets whose tumor-promoting roles are scaffold-mediated rather than catalytic, the fidelity of the degradation tool, not only its efficiency, is a critical determinant of whether chemogenetic models faithfully predict the biological outcome.

## Materials and Methods

### Mouse models and ethics

The following genetically engineered alleles were used for this study: *Kras*^LSLG12V^, *Trp53*^L^ (Marino et al, 2000), and *dTAG-Raf1*^KI^ (this work). Generation of the *Kras*^LSLG12V^ allele followed procedures similar to those previously described for *Kras*^LSLG12Vgeo^ (Guerra et al, 2003).

All mice used in this work were housed in specific pathogen-free conditions in the Animal Facility of the Spanish National Cancer Research Centre (AAALAC, JRS: dpR 001659), in accordance with Federation of European Laboratory Animal Science Association (FELASA) recommendations and following European Union legislation. Mice were subjected to 12-h light/dark cycles in ventilated racks under controlled conditions of temperature and humidity. Animals were housed in groups of 4–5 per cage with environmental enrichment. Animals had access to sterilized tap water and chow ad libitum. They were fed with a standardized diet. Both female and male mice were used for the experiments. Health status and body weight were monitored three times per week by the researchers and daily by the animal facility care-takers, ensuring early detection of any signs of distress or disease.

A total of 144 animals were included in the study. 12 tumor-free dTAG-*Raf1*^KI/KI^ mice were used for the systemic tolerability assessment and treated with dTAG^V^-1m (n = 4), vehicle (n = 4), or IGP002 (n = 4) for 30 consecutive days.

For tumor studies, 87 mice were infected intranasally with 1 × 10^6^ plaque-forming units (pfu) AdCre and 45 with 1 × 10^7^ pfu. Of these, eight mice reached the predefined *humane end point* before contributing to any analysis and were excluded. Six additional mice from the 1 × 10^7^ pfu cohort died during or shortly after infection. The remaining mice contributed to tumor onset statistics (Fig 1D and E). Five mice from the 1 × 10^6^ pfu cohort were used to establish primary LUAD cell lines before treatment. Four mice were found dead in cage before treatment initiation, most probably because of respiratory dysfunction. The remaining tumor-bearing mice were randomly assigned to treatment groups: dTAG^V^-1m (n = 7), vehicle (n = 5), or IGP002 (n = 3). IGP002-treated mice were excluded from efficacy analysis after detection of off-target FKBP12 degradation.

Criteria for determining *humane end point* included loss of more than 20% of the initial body weight, abnormal activity, abnormal physical appearance (fur, skin, posture, etc.), and signs of respiratory malfunction (dyspnea, rales, and extensive atelectasis detected by CT measurement). Once animals showed the above-mentioned symptoms, they were euthanized within the following 10 min. Mice were euthanized by CO_2_ inhalation according to the guidelines of the Ethics and Animal Welfare Committee (CEyBA).

All personnel involved in the study were adequately trained and certified in laboratory animal science, meeting the educational and competency standards recommended by FELASA to ensure responsible and ethical handling of animals throughout the research process. All experiments were approved by the Ethical Committees of the CNIO, the Carlos III Health Institute, and the Autonomous Community of Madrid (PROEX 119/17).

### Generation of the dTAG-Raf1 knock-in allele

The endogenous *Raf1* locus was modified using a targeting vector (Gene Bridges GmbH) containing the FKBP12^F36V^ coding sequence preceded by a double HA tag, inserted into exon 2. The vector was linearized with AscI and electroporated into G4 embryonic stem (ES) cells. Recombinant clones were verified by Southern blot using genomic DNA digested with HindIII (left arm) or EcoRV (right arm) and hybridized with external probes located ∼4.5 kb upstream (627 bp) and downstream (589 bp) of exon 2. Three independent ES clones were microinjected into C57BL/6J blastocysts to generate chimeric mice. Male chimeras were bred with 129Sv/J females to obtain germline transmission. The NeoR/KanR cassette was excised by fertilizing oocytes from *dTAG-Raf1*^+/KI^ females with sperm from *Tg.CAG-Flp*^T/T^ males. Complete cassette deletion was confirmed by genotyping PCR. All electroporation, microinjection, and genotyping procedures were carried out by the CNIO Transgenic Mice Unit.

### Tumor induction and treatment

LUADs were induced in 8- to 10-week-old anesthetized mice. Anesthesia was administered via an intraperitoneal injection of ketamine (75 mg/kg) and xylazine (12 mg/kg). Throughout the entire process, the eyes were protected with an ophthalmic ointment (Lacryvisc) to prevent corneal drying. Tumors were initiated by intranasal instillation of a single dose of 10^6^ pfu of adenovirus expressing optimized CRE recombinase (AdCre). This induced the expression of *Kras*^G12V^ and loss of *Trp53*, leading to multifocal LUADs detectable by CT imaging.

Tumor-bearing mice meeting the inclusion criteria were randomly assigned to treatment groups. CT-based tumor volume quantification was performed blinded to treatment group. Mice with CT-confirmed tumors (≥5 mm^3^) received daily intraperitoneal injections of dTAG^V^-1m (40 mg/kg), synthesized as previously described (Martín et al, 2026). The vehicle consisted of 5% DMSO and 20% Kolliphor EL (Cat. 61791-12-6; Sigma-Aldrich) in saline. The treatment lasted 2 mo.

### In vivo imaging and tumor volume quantification

Tumor volumes were measured using a SuperArgus CompaCT scanner (Sedecal) under 4% isoflurane anesthesia (Braun Vetcare). Image processing, analysis, and 3D rendering were performed using the 3D Slicer Viewer Software. Volumes were calculated assuming ellipsoid geometry:

$$V\approx \left(d_{long}\times d_{short}\times d_{short}\right)/2.$$

Mice were monitored daily for health status, and CT imaging was repeated every 4 wk to quantify tumor volume changes. Mice were euthanized at the end of the experiment or if they reached the *humane end point*—loss of more than 20% of the initial weight, anorexia, abnormal activity, abnormal physical appearance, and signs of respiratory distress.

### Systemic tolerability assessment

#### Densitometry

Densitometry scans were performed every 15 d using a Lunar PIXImus densitometer (GE Healthcare) by the Molecular Imaging Unit at CNIO. Mice were anesthetized with 3% isoflurane in oxygen at a flow rate of 0.5 liters/min.

#### Echocardiography

Echocardiographic assessments were conducted every 15 d. Anesthesia and thermal regulation were as described for densitometry. A Vevo 3100 micro-ultrasound system (VisualSonics) equipped with a 40-MHz transducer (RMV704) was used. Measurements included the left ventricular internal diameter in systole (LVIDs) and diastole (LVIDd). Left ventricular volumes in systole (LVVs) and diastole (LVVd) were calculated using the Teichholz formula:$\mathrm{LVV}\;({\mathrm{mm}}^{3})\;=\;[7/(2.4\;+\;\mathrm{LVID})]\;\times \;{\mathrm{LVID}}^{3}.$ Ejection fraction (EF) and fractional shortening (FS) were calculated as: EF (%) = [(LVVd − LVVs)/LVVd] × 100; FS (%) = [(LVIDd − LVIDs)/LVIDd] × 100.

#### Hematology

Blood was collected via facial vein (in vivo) or cardiac puncture (ex vivo). Complete blood counts were performed using a LaserCell blood counter (CVM Diagnóstico Veterinario SL), including total and differential white blood cells, lymphocytes, red blood cells, hemoglobin, and hematocrit.

### Necropsy, histopathology, and IHC

Necropsies were carried out in the dissection laboratory at CNIO, involving euthanasia of mice in a CO_2_ chamber (5-min cycle). Death was confirmed by cervical dislocation before tissue harvesting. A comprehensive approach was followed to collect tissue samples, which were then subjected to various preservation methods based on experimental requirements. Specifically, samples were taken in 10% formalin, for subsequent inclusion in paraffin blocks, and cut and stained as needed by the Histopathology Unit at CNIO. In addition, partial tissue samples were collected in microcentrifuge tubes and snap-frozen in dry ice-cooled 2-methylbutane for the subsequent extraction of DNA, RNA, or protein.

For histopathological visualization, tissue sections of 2.5 μm thickness were stained with hematoxylin & eosin (H&E). For IHC staining, a rat monoclonal α-RAF1 (1:200, Ref. EMI411E; Monoclonal Antibodies Unit) was used. Both H&E and IHC-stained sections were scanned using the Axio Scan.Z1 scanner (Zeiss). Visualization and analysis were performed with QuPath v0.5.1. Histopathological assessment was performed blinded to treatment group.

### Cell models and culture conditions

Cell lines used in this study included low passage immortalized *hUBC-CreERT2*^T^*; Araf*^L/Y^; *Braf*^L/L^; *Raf1*^L/L^ (RAFless) MEFs, a long-established conditional RAF-deficient model generated in the Barbacid laboratory and used in previous studies (García-Alonso et al, 2022; Paniagua et al, 2022) and murine epithelial cell lines established from *Kras*^G12V^; *Trp53*^null^ LUAD. The tumor-derived LUAD cell models included *dTAG-RAF1*^KI^ cells (generated in this work) and conditional *Raf1*^L^ cells expressing *hUB-CreERT2* (Sanclemente et al, 2018).

Cell lines were maintained at 37°C in a humidified atmosphere containing 5% CO_2_ and 16% O_2_ in Dulbecco’s Modified Eagle’s Medium (DMEM; Ref. L0103; Biowest) supplemented with 10% (vol/vol) fetal bovine serum (FBS; Ref. A5256701; Gibco), and 1% (vol/vol) penicillin–streptomycin (Ref. 15140122; Gibco). Raf1-deleted cells were additionally maintained in the presence of 600 nM 4-OHT.

### Establishment of tumor-derived LUAD cells

Tumor-derived cell lines were established from individual LUADs from mice of the indicated genotypes. After euthanasia, the lungs were collected in PBS supplemented with penicillin–streptomycin. Individual tumors were isolated under sterile conditions, mechanically minced with scalpels and transferred to complete culture medium. The medium was replaced every 2–3 d until stable adherent cultures were established. Cell lines were cryopreserved in DMEM supplemented with 10% DMSO and 10% FBS.

The identity and *Raf1* allele status of the resulting cultures were verified by genotyping PCR and by immunoblotting for RAF1 or dTAG-RAF1, including confirmation of the expected response to 4-OHT or dTAG^V^-1m.

### Lentiviral transduction and RAFless rescue assay

Lentiviral particles were produced in HEK293T/17 cells by co-transfection of 5 μg of the indicated expression vector with 1.95 μg pLP1, 1.3 μg pLP2, and 1.64 μg pLP/VSVG using polyethylenimine. Viral supernatants were collected 48 h after transfection, filtered through a 0.45-μm filter, and stored at −80°C.

RAFless MEFs were transduced at low confluence in the presence of 10 μg/ml polybrene and subsequently selected with 4 μg/ml puromycin. Cells were maintained as antibiotic-selected polyclonal populations, and no individual clones were isolated. Before the rescue assay, endogenous RAF proteins were eliminated by Cre-mediated recombination. RAF ablation was subsequently maintained by supplementing the culture medium with 600 nM 4-OHT throughout all experiments to prevent re-expression from unrecombined alleles in escaper cells. Then, to assess the ability of the indicated RAF constructs to rescue proliferation in the absence of endogenous RAF proteins, RAFless MEFs were transduced with lentiviral particles encoding WT RAF1, dTAG-RAF1, or empty vector, as indicated. After antibiotic selection, 10,000 cells per 100 mm dish were seeded in DMEM supplemented with 10% (vol/vol) FBS and cultured for 10–14 d with medium replacement every 3–4 d. In cells expressing dTAG-RAF1, dTAG^V^-1m was added at a final concentration of 500 nM and refreshed every 2 d to maintain sustained protein degradation throughout the assay. At the end point, the colonies were fixed with 4% paraformaldehyde for 15 min at room temperature, washed twice with PBS, and stained with 0.5% (wt/vol) crystal violet (Sigma-Aldrich).

### Apoptosis induction assays

Apoptosis induction assays were performed in primary LUAD cell lines derived from *Kras*^+/G12V^; *Trp53*^−/−^; *Raf1*^L/L^ or *Kras*^+/G12V^; *Trp53*^−/−^; *dTAG-Raf1*^KI/KI^ tumors, as indicated. Cells were treated with either vehicle, 50 μM etoposide, 300 nM dTAG^V^-1m, or a combination of both drugs for 24 h. Cells were detached, counted, and 200,000 cells per condition were collected in 200 μl of media. Apoptosis was assessed by using the FITC Annexin V Apoptosis Detection Kit I (#556547; BD Biosciences), following the manufacturer’s instructions. Samples were analyzed by flow cytometry in a Gallios device (Beckman Coulter). Annexin V-FITC and propidium iodide staining were used to distinguish viable, early apoptotic, and late apoptotic/necrotic cell populations, and data were analyzed and plotted using GraphPad.

For Western blot analysis, cells were treated with either vehicle, 50 μM etoposide, 300 nM dTAG^V^-1m, or a combination of both drugs for 24 h. Cell pellets were collected, washed with cold PBS, and lysed with 8 M urea containing 1% CHAPS with constant shaking at 4°C for 1 h. Lysates were centrifuged at 17,000*g* for 10 min at 4°C and the supernatants were collected for protein quantification. For the electrophoresis, 20 μg of protein with loading buffer (NuPAGE LDS sample buffer, 10% β-mercaptoethanol) was denatured for 10 min at 70°C, separated on 4–12% SDS–PAGE gels (Invitrogen), and transferred into nitrocellulose membranes using the Trans-Blot Turbo Transfer System. Membranes were blocked in 5% (wt/vol) nonfat powder milk/TBS-T or 5% BSA Albumin Fraction V (Ref.A6588.0100; Panreac Quimica, S.L.U.) for 1 h and later incubated at 4°C overnight with the primary antibodies in TBS-T. Next day, the membranes were washed twice for 5 min in TBS-T and then incubated with the secondary antibodies at RT for 1 h. Enhanced chemiluminescence measurement was performed in a ChemiDoc MP Imaging system. The primary antibodies used are the following: β-ACTIN (A5441; Sigma-Aldrich, 1:20,000), PARP (9542; Cell Signaling, 1:1,000), RAF1 (53745; Cell Signaling, 1:1,000), and CASP3 (9662; Cell Signaling, 1:1,000). The secondary antibodies used are the following: anti-rabbit (115-035-003) and anti-mouse (111-035-003) from Jackson ImmunoResearch (1:5,000 dilution).

### RNA extraction and qRT-PCR

Total RNA was purified using the RNeasy Mini Kit (Ref. 74104; Qiagen), including on-column DNase digestion with the RNase-Free DNase Set (Ref. 79254; Qiagen), according to the manufacturer’s instructions. RNA concentration was determined using a NanoDrop spectrophotometer (Thermo Fisher Scientific). For cDNA synthesis, 1 μg of RNA was reverse-transcribed using SuperScript II Reverse Transcriptase (Ref. 18064022; Invitrogen) and random primers. The resulting cDNA was diluted 1:20 and analyzed using SYBR Green PCR Master Mix (Ref. 4367659; Applied Biosystems) with primers specific for *Raf1* (forward: 5′-AAATTAGGCCTCGTGGGCAG-3′; reverse: 5′-TGCAACATCTCCATGCCACT-3′) and *Rn18s* (forward: 5′-GTAACCCGTTGAACCCCATT-3′; reverse: 5′-CCATCCAATCGGTAGTAGCG-3′) in a QuantStudio 6 Flex Real-Time PCR System. Reactions were performed in technical triplicates using the following cycling conditions: 20 s at 95°C, followed by 40 cycles of 1 s at 95°C and 20 s at 60°C, and a final melting-curve analysis.

Relative *Raf1* mRNA expression was calculated using the comparative threshold-cycle method. For each sample, *Raf1* Ct values were normalized to the endogenous housekeeping gene *Rn18s* (18S rRNA) according to ΔCt = Ct_*Raf1* − Ct_*Rn18s*. The mean ΔCt of the *Raf1*^L/L^ control group was used as the reference, and ΔΔCt was calculated as ΔCt_sample − mean ΔCt_*Raf1*^L/L^. Relative fold expression was calculated as 2^−ΔΔCt^. Data in Fig 1C are represented as −ΔΔCt, which corresponds to the log_2_-transformed relative expression. Accordingly, a value of zero indicates no change relative to the control group, whereas positive and negative values indicate increased and decreased expression, respectively.

### Kinase assay

To assess RAF1-dependent phosphotransferase activity, protein complexes were expressed in Expi293F cells by co-transfection of pcDNA3-HA-HSP90 (Ref. 22487; Addgene), pCMV-CDC37-myc-DDK (Ref. RC201002; Origene), and the indicated Strep-tagged RAF1 construct (pcDNA3-2xHA-FKBP12^F36V^-RAF1-Strep, pcDNA3-RAF1-Strep, or pcDNA3-RAF1^Y340E/Y341E^-Strep). 48 h post-transfection, the cells were lysed in buffer containing 20 mM Tris–HCl, pH 7.5, 150 mM NaCl, 10 mM MgCl_2_, 10 mM KCl, 20 mM Na_2_MoO_4_, and 0.1% Triton X-100, supplemented with protease and phosphatase inhibitor cocktails and Benzonase. Clarified lysates were purified by affinity chromatography using Strep-Tactin Sepharose resin (Ref. 2-1201-010; IBA) and eluted with washing buffer supplemented with 10 mM desthiobiotin (Ref. 2-1000-002; IBA). In vitro kinase reactions were performed in ADBI buffer (20 mM MOPS, pH 7.2, 5 mM EGTA, 1 mM Na_3_VO_4_, and 1 mM DTT) supplemented with ATP and Mg^2+^, using identical amounts of recombinant MEK1 (rMEK; Ref. 14-420; Sigma-Aldrich) as substrate at 1 μg per reaction. Constitutively active recombinant RAF1 (rRAF1; Ref. R1656; Sigma-Aldrich) was included as a positive control. Reactions were incubated at 30°C for 30 min and analyzed by Western blotting.

### Immunofluorescence

Cells were fixed with 4% paraformaldehyde (Ref. 28908; Thermo Fisher Scientific) for 10 min at room temperature and permeabilized with 0.2% Triton X-100 in PBS for 10 min. Blocking was performed in PBS containing 1% BSA and 0.1% Tween-20 for 1 h at room temperature. Primary antibodies α-RAF1 (sc-7267; Santa Cruz, 1:100) and α-HA (3724S; Cell Signaling Technology, 1:500) were incubated overnight at 4°C, followed by Alexa Fluor 488–conjugated α-mouse (A11001; Invitrogen, 1:250) or Alexa Fluor 647–conjugated α-rabbit (A31573; Invitrogen, 1:250) secondary antibodies for 1 h at room temperature. Images were acquired using a LIPSI high-content screening system (Nikon) with a 40× objective.

### Western blotting

Protein lysates were prepared in lysis buffer (50 mM Tris–HCl, pH 7.5, 150 mM NaCl, and 0.5% NP-40) with protease and phosphatase inhibitors. Equal protein amounts (30 μg) were resolved on NuPAGE 4–12% Bis-Tris gels (Invitrogen), transferred to nitrocellulose membranes, and probed with antibodies against: ARAF (4432; Cell Signaling), BRAF (sc-5284; Santa Cruz), RAF1 (610151; BD Biosciences), Vinculin (V9131; Sigma-Aldrich), CASP3 (9662; Cell Signaling), cl.CASP3 (9661; Cell Signaling), and PARP (9542; Cell Signaling). For detection of total MEK1/2, antibodies against MEK1 (sc-6250; Santa Cruz) and MEK2 (610235; BD Biosciences) were combined during membrane incubation. Similarly, antibodies against ERK1 (554100; BD Biosciences) and ERK2 (610103; BD Biosciences) were combined for detection of total ERK1/2. Phosphorylated MEK1/2 and ERK1/2 were detected using antibodies that recognize both phosphorylated isoforms (9154 and 9101; Cell Signaling, respectively).

### Immunoprecipitation and LC-MS/MS analysis

Interactome analyses were performed using one *Kras*^G12V^/*Trp53*^null^ LUAD-derived cell line from each of three RAF1 conditions: a RAF1-proficient line derived from conditional *Raf1*^L/L^ tumors (Sanclemente et al, 2018), an RAF1-null line derived from the same conditional model after Raf1 recombination (Sanclemente et al, 2018), and a homozygous *dTAG-Raf1*^KI/KI^ line established in this study. The latter expresses dTAG-RAF1 from the endogenous Raf1 locus and does not express native WT RAF1.

Five IP-MS conditions were analyzed, with two replicate samples per condition: anti-RAF1 immunoprecipitation from RAF1-proficient cells (C1), anti-RAF1 immunoprecipitation from *dTAG-Raf1*^KI/KI^ cells (C2), anti-RAF1 immunoprecipitation from RAF1-null cells (C3), anti-HA immunoprecipitation from *dTAG-Raf1*^KI/KI^ cells (C5), and anti-HA immunoprecipitation from HA-negative RAF1-proficient cells (C6). The primary WT RAF1 versus dTAG-RAF1 interactome comparison was C2 versus C1 and therefore used the same anti-RAF1 affinity reagent in both conditions. C3 and C6 served as negative controls for the anti-RAF1 and anti-HA pulldowns, respectively, whereas C2 versus C5 was used to assess affinity reagent–dependent protein recovery.

Cells in 15 cm dishes were lysed in ice-cold IP buffer (50 mM Tris–HCl, pH 7.5, 150 mM NaCl, 10% glycerol, 1% Triton X-100, and 1 mM EDTA + inhibitors). 200 μg of protein was incubated overnight at 4°C with either α-RAF1 (53745S; CST) or α-HA magnetic beads (SAE0197; Sigma-Aldrich). Beads were washed and resuspended in 50 mM ammonium bicarbonate for on-bead digestion with trypsin. Peptides were acidified with 1% formic acid, cleaned on C18 stage-tips, fractionated on SCX columns, and reconstituted in 3% ACN, 1% FA for LC-MS.

Peptides were analyzed on an Orbitrap Eclipse Tribrid MS (Thermo Fisher Scientific) via an EVOSEP One interface. Separation was performed on a 15 cm × 150 μm C18 column (1.5 μm beads), using an 88-minute DIA gradient (15 samples/day) (Bache et al, 2018). MS1 scans (m/z 350-1,200) were acquired at 120,000 resolution; MS2 scans used 33 DIA windows at 30,000 resolution. HCD fragmentation used 28% normalized collision energy. Data were acquired using Tune v3.5.3890 and Xcalibur v4.5.

### Proteomic and interactome analysis

Raw data were analyzed using DIA-NN (v1.8.2 beta 27) in library-free mode (Demichev et al, 2020). Searches were run against the *Mus musculus* UniProt database (February 2024), with custom entries for dTAG-RAF1 and common contaminants. Variable modifications included N-terminal acetylation, methionine oxidation, and methionine excision. MaxLFQ-based quantification (Cox et al, 2014) was performed via the diann-r package in R (v4.0.1). Proteins were filtered by q-value (<0.01), peptide count, and missingness. Differential expression was analyzed using limma (Ritchie et al, 2015); significance was defined as |FC| > 1.5 and adjusted *P* < 0.05.

Interactors were filtered based on cytosolic localization (GO and Human Protein Atlas [Thul et al, 2017]) and cross-referenced with BioGRID (Oughtred et al, 2021) and HuRI (Luck et al, 2020) (accessed May 2024). Pathway enrichment was conducted in R using clusterProfiler (Yu et al, 2012) (v2023.12.1) with Reactome annotations (Milacic et al, 2024) and FDR < 0.05. A secondary enrichment analysis using the Reactome database annotations was restricted to cell death–related terms. Visualizations were generated using enrichplot and heatmaps via seaborn (Waskom, 2021) in Python (v3.12.4).

Protein-group abundances were quantified using MaxLFQ and were not calculated from raw peptide or spectral counts. For differential-abundance analysis, MaxLFQ values were log_2_-transformed and analyzed using limma. The primary interactome contrast was C2 versus C1. Proteins significantly differing between the anti-RAF1 and anti-HA pulldowns from *dTAG-Raf1*^KI/KI^ cells (C2 versus C5), but not in any biological or negative-control contrast, were considered potentially affinity reagent dependent and excluded from subsequent analyses. For the normalized heatmap shown in Fig 5A, interactor log_2_ fold changes were expressed relative to the RAF1 bait log_2_ fold change. Both absolute and RAF1-normalized fold changes are presented.

### Exploratory ubiquitination site compilation and structural annotation

To compile a comprehensive RAF1 ubiquitination profile, data were integrated from three independent sources: experimentally validated sites retrieved from curated repositories (PhosphoSitePlus), computationally predicted sites generated using the RUBI and UbiSite *in silico*, and lysine-GlyGly (K-ε-GG) peptides identified across three large-scale ubiquitinome datasets (Udeshi et al, 2020; Hansen et al, 2021; Rivera et al, 2021).

For structural analysis, the identified lysines were mapped onto available three-dimensional models of RAF1 using PyMOL (v3.0, Schrödinger LLC). Structures used included RAF1 models from the PDB (3OMV and 7Z38). This analysis was used for structural annotation of reported RAF1 lysine ubiquitination sites and was not intended to predict lysines productively modified upon degrader-induced E3 ligase recruitment.

### Statistical analysis

All statistical analyses were performed with GraphPad Prism (v8.4.0) or R (v4.4.2). Data are presented as mean ± SEM. For two-group comparisons, unpaired *t* test (parametric) or Mann–Whitney U test (nonparametric) were used. Longitudinal tumor data were analyzed with linear mixed-effects models fitted by REML, which leverage repeated measurements per animal to improve statistical resolution given the small cohort size, a direct consequence of the reduced tumor penetrance of the *dTAG-Raf1*^KI/KI^ model. Significance was defined as: *P* < 0.05 (*), < 0.01 (**), < 0.001 (***), and < 0.0001 (****).

Sample sizes were estimated based on effect sizes and variances observed in previous studies from this laboratory using the same GEMM (Sanclemente et al, 2018, 2021). No formal power calculation was performed.

## Supplementary Material

Reviewer comments

## Data Availability

The mass spectrometry proteomics data generated in this study have been deposited in the ProteomeXchange Consortium via the PRIDE partner repository (Perez-Riverol et al, 2022) under accession number PXD079461. All other data supporting the findings of this study are available within the article and its supplementary information.
