# Supplementary material for: RAF1 scaffold integrity shapes chemogenetic degradation outcomes in KRAS-driven lung cancer models
Source: Life Sci Alliance. 2026 Sep 22;9(12):e202603839. doi: 10.26508/lsa.202603839 (PMC13598765; doi:10.26508/lsa.202603839)
Supplement: Supplementary file 1 [file LSA-2026-03839_review_history.pdf]

RAF1 scaffold integrity shapes chemogenetic degradation outcomes in KRAS-driven lung cancer models

Laura de-la-Puente-Ovejero, Ana Domostegui, Inés García-Pérez, Gonzalo Aizpurua, Lucía Lomba Riego, Pilar Ximénez-Embún, Cristina Mayor-Ruiz, Mariano Barbacid, and Sara García-Alonso
DOI: https://doi.org/10.26508/lsa.202603839

Corresponding author(s): Sara García-Alonso, Spanish National Cancer Research Centre and Mariano Barbacid, Centro Nacional de Investigaciones Oncológicas (CNIO)

**Review Timeline:**		
	Submission Date:	2026-07-07
	Editorial Decision:	2026-08-24
	Revision Received:	2026-08-31
	Accepted:	2026-09-02

Scientific Editor: Sarita Hebbar

Transaction Report:

(Note: With the exception of the correction of typographical or spelling errors that could be a source of ambiguity, letters and reports are not edited. The original formatting of letters and referee reports may not be reflected in this compilation.)

August 24, 2026

RE: Life Science Alliance Manuscript #LSA-2026-03839-T

Sara García-Alonso
Centro Nacional de Investigaciones Oncológicas
Tumor Biology Programme
Melchor Fernández Almagro 3
Madrid, Madrid 28029
Spain

Dear Dr. García-Alonso,

Thank you for submitting your revised manuscript entitled "RAF1 scaffold integrity shapes chemogenetic degradation outcomes in KRAS-driven lung cancer".

We apologise for the delay in communicating our decision due to reviewer and editor availability issues. Now your manuscript has been reviewed by three expert reviewers whose comments are appended below. As you will note, all the reviewers have commented positively on your study and the importance of these findings.
Reviewer 1 and 3 are clearly supportive of this work. Reviewer 2, whilst positively commenting on the design and data, has indicated the need for experimental evidence regarding the effect of N-terminal tag on HSP90-CDC37 complex, functional relevance of the structural analysis, additional controls. Based on the overall support from Reviewers 1 and 3, we would be happy to publish your paper in Life Science Alliance without further experiments, but pending resolution of the following points from the reviewers as well as final revisions necessary to meet our formatting guidelines.

-We agree with the multiple points from Reviewers 2 and 3 that the results must be accurately described, and all the methods must be completely described.
-We concur with all the reviewers that the discussion section must be expanded to elaborate on the relevance of the structural studies in terms of future efforts, and to expand on implications for earlier studies.
-In line with comments from the reviewers, we agree that you must tone down the novelty claim for the impact of a tag on protein function and limit the focus throughout the ms to conclusions on experimental models studied in this work.

Please include a point-by-point response to all the reviewers' concerns and provide a revised manuscript with the changes highlighted.

MANUSCRIPT ORGANIZATION AND FORMATTING:

To avoid unnecessary delays in the acceptance and publication of your paper, please read the following information carefully. Full guidelines are available on our Instructions for Authors page, https://www.life-science-alliance.org/authors

Submission of a paper that does not conform to Life Science Alliance guidelines will delay the acceptance of your manuscript.

-Please include molecular weight markers for all images of blots.
-Kindly include details of objectives used in imaging experiments.
-Please add ORCID ID for corresponding (and secondary corresponding) author--you should have received instructions on how to do so.
-Please add a Summary Blurb/Alternate Abstract, Category, and Keywords for your manuscript in our system.
-Please add the X and Bluesky handles of your host institute/organization, as well as your own, and/or one of the authors, in our system.
-Please be sure that names of the authors match between the system and manuscript file.
-Please add authors' contributions to our system as well.
-Please rename "Disclosure and competing interests statement" to "Conflict of Interest".
-LSA allows supplementary figures, but not EV Figures; please update legends, labels, and callouts for the Supplementary Figures in the manuscript Fig EV1A = Fig S1A).
-Please be sure that the authorship listing and order is correct

We encourage our authors to provide original source data, particularly uncropped/-processed electrophoretic blots and spreadsheets for the main figures of the manuscript. If you would like to add source data, we would welcome one PDF/Excel-file per figure for this information. These files will be linked as supplementary "Source Data" files.

It is Life Science Alliance policy that if requested, original data images must be made available to the editors. Failure to provide original images upon request will result in unavoidable delays in publication. Please ensure that you have access to all original data images prior to final submission.

If you are planning a press release on your work, please inform us immediately to allow informing our production team and scheduling a release date.

We welcome submissions of potential cover images for the issue of LSA in which your work would appear. If you have high quality images associated with this work, please feel free to email these, with a caption, to the journal office.

LSA encourages authors to provide a 30-60 second video where the study is briefly explained. These videos will be appear embedded with the manuscript online at Life Science Alliance, and on social media to promote the published paper and authors (for examples, see https://docs.google.com/document/d/1-UWCfbE4pGcDdcgzcmiuJl2XMBJnxKYeqRvLLrLSo8s/edit?usp=sharing). Corresponding or first-authors are welcome to submit the video. Please submit only one video per manuscript. The video can be emailed to contact@life-science-alliance.org

FINAL FILES:

To upload the final version of your manuscript, please log in to your account: https://lsa.msubmit.net/cgi-bin/main.plex
You will be guided to complete the submission of your revised manuscript and to fill in all necessary information. Please get in touch in case you do not know or remember your login name.

The following items are required for acceptance.

-- An editable version of the final text (.DOC or .DOCX) is needed for copyediting (no PDFs).

-- High-resolution figure, supplementary figure and video files uploaded as individual files: See our detailed guidelines for preparing your production-ready images, https://www.life-science-alliance.org/authors

-- Summary blurb (enter in submission system): A short text summarizing in a single sentence the study (max. 200 characters including spaces). This text is used in conjunction with the titles of papers, hence should be informative and complementary to the title. It should describe the context and significance of the findings for a general readership; it should be written in the present tense and refer to the work in the third person. Author names should not be mentioned.

The license to publish form must be signed before your manuscript can be sent to production. A link to the license to publish form will be available to the corresponding author only. Please take a moment to check your funder requirements.

Reviews, decision letters, and point-by-point responses associated with peer-review at Life Science Alliance will be published, alongside the manuscript. If you do want to opt out of having the reviewer reports and your point-by-point responses displayed, please let us know immediately.

Thank you for your attention to these final processing requirements. Please revise and format the manuscript and upload materials as soon as you are able.

Thank you for this interesting contribution to the literature. We look forward to publishing your paper in Life Science Alliance.

Sincerely,

Sarita Hebbar, PhD
Scientific Editor
Life Science Alliance
http://www.lsajournal.org

Reviewer #1 (Comments to the Authors (Required)):

This is an important study which evaluates the consequences of N-terminal tag fusion and PROTAC-mediated degradation of RAF1, a potential TPD target and vulnerability in KRAS mutant tumours, using the dTAG system. The key finding is that the tag fusion impairs some aspects of RAF1 function even in the non-induced state, resulting in differences in tumour sensitivity between animals carrying tagged versus wildtype alleles even before protein degradation is induced. This is rationalised by showing that the tag has minimal effects on kinase function but does affect protein-protein interactions, consistent with previous results indicating that the scaffold function of RAF1 is critical for tumourigenesis. Degradation of dTAG-RAF1 has little impact on the progression of tumours that do emerge in this model, indicating that degradation of a subfunctional protein in this case does

not phenocopy the effect of genetic deletion.

Overall the study is well conducted and the conclusions are justified. It builds on a growing body of literature highlighting that the fusion of tags to proteins can impair their function, and that the consequences of tag fusion in vivo can be challenging to predict based on in vitro data. I believe the study is suitable for publication in its current form and have only one suggestion for the discussion.

For the benefit of other researchers hoping to avoid the types of problem described in this manuscript, I would encourage the authors to further develop their discussion of how their learnings might inform the allele design process. They compared C- and N-terminal tag fusion but there is theoretically much more that could be done to minimise the level of perturbation introduced by the tag. It is beyond the scope of the current study to explore this experimentally, and I acknowledge that this point is already touched upon in the current discussion (lines 287 - 290). But specifically how might the authors incorporate structural insights into future efforts to design a tagged RAF1 protein that retained scaffolding function? Are there strong candidate sites for internal fusion, and how might the authors prioritise them?

Reviewer #2 (Comments to the Authors (Required)):

This study investigates dTAG-mediated RAF1 degradation in a genetically engineered KRAS-driven lung adenocarcinoma model. Although pharmacological degradation of dTAG-RAF1 does not provide an apparent therapeutic benefit and fails to reproduce the tumour regression observed following genetic RAF1 ablation, the study nevertheless provides valuable insights into the design and interpretation of targeted protein-degradation approaches. In particular, the finding that introduction of a degradation tag may itself alter the biological properties of the target protein represents an important methodological consideration with relevance beyond RAF1. Overall, the study is carefully designed, supported by substantial in vivo and cellular experimentation, and the data are generally clearly presented and figures are generally clearly presented. However, several experimental and mechanistic aspects require clarifications to fully support their main conclusions.

- The authors appear to argue that the N-terminal FKBP12F36V tag perturbs RAF1 scaffold functions. At the same time, RAF1 is also maintained in a specific conformational state by the HSP90-CDC37 complex acting on the kinase domain. It would therefore be informative to determine whether the N-terminal tag alters this complex and whether changes in chaperone occupancy could indirectly remodel the RAF1 interactome and contribute to the altered scaffold behaviour observed.

- The structural analysis of RAF1 ubiquitination sites appears disconnected from the central mechanistic story, as no experimental evidence links K462/K575 to dTAG-mediated degradation or the altered scaffold function of tagged RAF1. This section therefore adds unnecessary complexity and could be moved to the Extended View or omitted unless its functional relevance is demonstrated.

- The authors state that the Raf1L/L and Raf1−/− data derive from historical cohorts generated in their laboratory. Were these data previously published? If so, the original study should be cited. Authors should explicitly acknowledge here (not only later in the Discussion) that these cohorts were generated and analysed at different times and provide sufficient information to assess their comparability, including genetic background, age/sex distribution, tumour-induction protocol, imaging schedule and experimental endpoints. These historical data should not be presented as directly equivalent to current study control groups unless such comparability can be demonstrated.

- Fig. 1: The authors attribute the altered functionality of dTAG-RAF1 specifically to the N-terminal FKBP12F36V part. However, the dTAG-RAF1 construct also contains an N-terminal 2×HA tag. An appropriate matched control (e.g. 2×HA-RAF1) would be the ideal one to distinguish effects caused by FKBP12F36V from those resulting from N-terminal tagging per se. 1B. To compare the RAF1 levels the samples should have been analysed on the same membrane under identical exposure conditions, with appropriate quantification. Separate blots cannot establish comparable protein abundance reliably. In addition, 4-OHT-induced recombination/RAF1 depletion should be demonstrated alongside the presented blots to verify that RAF1 deletion occurred. 1D-E. The choice of the historical Raf1L/L cohort as the comparator requires further justification. Differences in genetic background and experimental conditions between the dTAG and historical RAF1L/L cohorts could otherwise contribute to the striking difference in tumour penetrance/onset.

- Fig. 2. The figure legend should explicitly state whether error bars represent SD or SEM, as these provide substantially different visual representations of variability. Although the Statistics section indicates that data are generally presented as mean {plus minus} SEM, variability differs markedly among panels. But looking at error bars e.g., A versus B, high variations are visible. For these physiological measurements, showing individual data points together with mean {plus minus} SD would provide a more informative representation of biological variability.
B-D. The Day 0 baseline data require clarification. The same animals appear to show relatively limited variability in body weight at Day 0, whereas fat mass and BMD show substantially greater and, importantly, markedly different dispersion between the vehicle and dTAGV-1m groups before treatment has begun. Please show the individual animal values, specify whether the error bars represent SD or SEM, and provide the sex distribution of each group. For these longitudinal measurements, connecting

individual animals across time would also substantially improve interpretation. E-F. Please clarify the meaning of the different symbols (triangles in E and circles in F).

- Fig. 3A-B. Since RAF1 degradation does not reduce MEK1/2-ERK1/2 phosphorylation, the possibility of compensatory signalling through the other RAF isoforms should be addressed. The activation/phosphorylation status of RAF proteins, particularly BRAF and RAF1 where appropriate, should be analysed, especially when talking about these RAFs in the proposed model about compensation. Fig. 3C-E. Although the numbers of mice and tumours analysed are provided, the presentation is somewhat difficult to follow because the number of tumours varies considerably between analyses. The authors should clearly indicate how many tumours originate from each individual mouse and, where possible, identify tumours belonging to the same animal in the plots. This is particularly important because multiple tumours derived from the same mouse cannot be considered fully independent biological replicates and the hierarchical structure of the data should be appropriately accounted for in the statistical analysis.

- Fig. 4A. Appropriate controls are required to establish that the observed PARP and caspase-3 cleavage results specifically from Raf1 deletion rather than prolonged 4-OHT exposure. In addition, the strongest apoptotic phenotype following genetic Raf1 deletion is observed at 96 h, whereas dTAGV-1m-treated cells are analysed only up to 72 h. A matched 96-h dTAGV-1m time point is required before concluding that acute RAF1 degradation fails to reproduce the apoptotic phenotype caused by genetic deletion. Some literature data indicate that 4-OHT itself may induce apoptotic/cell-death responses (PMID: 23722551). Given the prolonged treatment (up to 96 h), such effects cannot be excluded, particularly since the 4-OHT concentration used is not clearly provided in the Methods. An appropriate 4-OHT-treated control in which Raf1 cannot be recombined should therefore be included to distinguish potential treatment-related effects from apoptosis specifically caused by RAF1 depletion. 4B. This experiment was performed only once and therefore cannot support a quantitative conclusion regarding apoptosis. The Annexin-V/PI analysis should be repeated in at least three independent biological experiments, with quantification and appropriate statistical analysis. 4C. The flow-cytometry gating strategy is not provided. Representative Annexin V/PI plots together with the complete gating strategy, including cell/singlet selection, compensation and the controls used to establish the Annexin V/PI gates, should be included. This is particularly important because these experiments are used to support the central conclusion that dTAG-mediated RAF1 degradation does not induce apoptosis.

Fig. 5. The interpretation of the proteomic data in relation to apoptosis is unclear. The authors propose that the N-terminal tag compromises RAF1-mediated anti-apoptotic scaffold functions. However, several proteins annotated as negative regulators of apoptosis appear more abundant in the dTAG-RAF1 interactome. How do the authors reconcile this enrichment with their proposed model? The directionality of the individual proteins contributing to the pathway enrichment should be presented and discussed rather than interpreting pathway enrichment alone as evidence for impaired anti-apoptotic RAF1 function.

- LUAD cell lines: The generation and characterization of the tumour-derived LUAD cell lines should be described in substantially greater detail. How many independent cell lines were established from individual tumours/mice, how were they authenticated/characterized, and how many independent lines were used for the experiments? EV3A-B appears to show only one representative line. If the conclusions in Fig. 4 are based predominantly on a single tumour-derived cell line, validation of the key findings in independently derived lines would substantially strengthen confidence in the conclusions.

- Cell models and reproducibility. The manuscript does not clearly state whether lentivirally generated cell lines represent polyclonal populations or individually selected clones, nor how many independently generated cell populations/clones were analysed. Since lentiviral transduction results in random genomic integration and potentially variable transgene copy number/expression, conclusions concerning functional differences between RAF1 constructs should not rely on a single independently generated line/clone. The authors should clarify how the cell lines were generated, report the number of independent clones/populations tested, and ideally demonstrate the key phenotypes in independently generated lines.

- Fig. 1C. It is unclear why Raf1 expression is presented as -ΔΔCt. Please clarify also the calculation in methods part.

- Fig. EV3E. Cells and nuclei in the upper panel appear considerably smaller than those in the two lower panels. Please clarify whether all images were acquired and displayed at the same magnification and whether the indicated scale bar applies equally to all panels. If the scale is indeed identical, is the apparent difference in cell/nuclear size a reproducible phenotype associated with the different RAF1 conditions?

- The study examines one scaffold protein, one N-terminal tag configuration and one degrader; the conclusions therefore cannot yet be broadly generalized to degron-based approaches. I would encourage the authors to soften the language accordingly in relevant parts of the manuscript.

- Row 158: "... suggesting that RAF1 is dispensable for normal tissue homeostasis". I consider that should not be generalised, should be used something like "under the conditions examined".

- The Discussion reads in several places as an extended summary of the Results. It would benefit from being streamlined and refocused on interpretation, limitations, alternative explanations and the broader implications of the findings.

- Methods/Western Blotting: Separate antibodies against MEK1, MEK2, ERK1 and ERK2 are listed in Materials/Methods, whereas the figures label signals as MEK1/2 and ERK1/2. Please clarify whether antibodies were combined for detection or whether a single isoform-specific antibody is being used to represent MEK1/2 or ERK1/2.

- IP/MS analysis: The Methods state that either anti-RAF1 or anti-HA magnetic beads were used for immunoprecipitation. Please specify which antibody was used for each condition. If WT RAF1 and dTAG-RAF1 interactomes were generated using different affinity reagents, the rationale and appropriate controls should be provided, as this may influence recovery of RAF1 and associated proteins.

Reviewer #3 (Comments to the Authors (Required)):

The work by de-la-Puente-Ovejero et al. describes the use of a chemogenetic approach to delete Raf1 in mice and explore its consequences and applicability as a therapeutic avenue in Ras-driven tumorigenesis. This follows up on a growing body of data originating from the authors´ and other laboratories pointing to Raf1 kinase inhibition as a promising therapeutic strategy for Ras driven cancers. Specifically, the authors generated a dTAG-RAF1 genetic mouse model and combined this with a previously well-established inducible KRasG12V system to induce lung adenocarcinoma (LUAD). Using this approach they show that Raf1 can be effectively removed chemogenetically without inducing overt toxicity in the animals, consistent with the results from genetic knockdown strategies applied previously by the same group and others. The chemogenetic deletion of Raf1 protein did however not significantly affect tumour growth, which was less pronounced in the dTAG-RAF1 animals in the first place. The authors conclude that the N-terminal appendage of the dTAG compromises protein-protein interactions that are essential to mediate the non-catalytic Raf1 function in curbing apoptosis. The authors substantiate this notion by a number of additional experiments including a proteomic comparative analysis of the RAF1 versus dTAG-RAF1 Interactome.
The study is well-designed and technically sophisticated. All of the experiments, as well as the interpretation and analysis of the data, are well-founded, sound and support the conclusions drawn. The finding that the chemogenetic deletion of Raf 1 is not toxic represents an important observation, given the promising prospects for RAF1 as a target molecule in Ras-dependent tumors. However, in my opinion, the central finding-that the dTAG-RAF1 fusion construct lacks a large portion of its anti-apoptotic functions due to the loss of scaffold integrity-is somewhat overrated in terms of novelty. This does however not negate the validity, soundness and relevance of the findings as the authors make a good job in rationalizing all findings around the mentioned scaffold integrity framework. I like also the final summary figure 6, which I believe is very helpful for the reader. Apart from that, I have no major issues or comments, as I believe that all the experimental data presented are fundamentally sound, justify the conclusions and will be of interest to readers in the fields of Ras/Raf and cancer research. At most, I would recommend that the authors tone down the novelty of the loss of scaffold integrity somewhat and place it in the correct context, as this essentially reflects the well-known fact that C- or N-terminal tags are likely to influence the function of the target protein - a fact that has long been recognised by researchers in the field of signal transduction. In addition, I have identified a number of minor points which I recommend the authors correct in a revised version of the manuscript:
• Since the study describes the effects of a Raf1 fusion protein on the integrity of the Raf1 scaffold, the conclusions primarily pertain to preclinical contexts. I would suggest limiting the focus and conclusions mainly to experimental models. The authors may consider also making this point clear in the title of the manuscript, for example, by editing such as this or something similar: RAF1 scaffold integrity shapes chemogenetic degradation outcomes in preclinical models of KRAS-driven lung cancer
• The results presented here also have numerous implications for earlier studies that used tagged Raf1. Many researchers, including this reviewer, have used N-terminally tagged Raf constructs in their studies, and some of these studies may need to be revisited. It might be helpful to briefly mention this fact.
• Immunoprecipitation and LC-MS/MS analysis: In my view, this part requires more experimental detail. The experiments were done in "in LUAD-derived cell lines" according to the main text. What cells exactly were used? Could the authors provide more detail in the experimental section? Why were different antibodies (HA vs. Raf1) used to pulldown WT Raf1 or dTAG-Raf1? Using different Abs for IP can affect the associated proteome differently, much along the argument of scaffold integrity made by the authors here in the paper. Also, was native WT RAF1 present in the dTAG-RAF1 cells used for the pulldown? In that case there would be a situation of competition for interactors between native and heterologous dTAG-RAF1. Would it not make sense to use the Rafless cells +/- dTAG-RAF1 allowing to pulldown WT Raf and dTAG-RAF1 with the same -Raf1 antibody to exclude all these confounders? Please specify and comment. Also I would suggest to provide more detail on the data analysis, e.g. were the peptide lists normalized internally? E.g. to the RAF1-bait peptide frequency?
• L77: „Second, the biological outcome of degradation depends not only on efficient target elimination, but also on whether loss of the protein reproduces the relevant functional consequences of genetic ablation." This sentence is ambiguous and its exact meaning is not clear to me.
• L86: "Indeed, emerging evidence suggests that proteins carrying chemogenetic tags may exhibit altered function even prior to degradation" This sentence illustrates my previous point: The fact that an attached tag can influence the function of a bait protein-whether it is a scaffold protein or another type of protein-is not a new finding, but has been known for many years. I suggest toning down these claims a little.
• Fig1A: The mouse model Is well illustrated but I would suggest to include further information e.g. in the figure legend, making clear to readers not familiar with Raf1 genetics that the ATG start codon resides in exon 2.
• L141: " ...in parallel, a second compound, IGP002 (a PROTAC analogue with improved solubility and metabolic..." Can the

authors please include a reference for IGP002?

- The Caption in Fig 3E is ambiguous and should be made more explicit to better describe the four groups/columns.
- In vitro kinase assays: Fig3D should include a load of rMEK by Coomassie or similar.
- The authors describe and document a lack of toxicity of Raf1 depletion, consistent with previous genetic Raf1 ablation data. This suggests that the apoptosis-regulatory role of Raf1 becomes only relevant in the context of oncogenic Ras signalling in tumorigenesis. Analogous selective effector engagement by oncogenic versus wt Ras has been suggested previously e.g. for PI3K (PMID: 30590030; PMID: 17540175; PMID: 30560710). All these data suggest that oncogenic Ras not just simply overactivates its effectors but rather it appears that it re-wires and re-purposes downstream effectors for oncogenesis. This is an intriguing implication which the authors might like to shortly comment on in the discussion part.
- Have the Rafless MEFs been published? Please specify. If not, please provide more background in the experimental part.
- Some information about the antibodies used for Western Blotting is confusing; e.g. The western image panels indicate pMEK1 while in the methods description a pMEK1/2 Ab is described. Please check and specify.
- Please indicate which cells were used in Fig 4. Were these MEFs?

Reviewer #1 (Comments to the Authors (Required)):

This is an important study which evaluates the consequences of N-terminal tag fusion and PROTAC-mediated degradation of RAF1, a potential TPD target and vulnerability in KRAS mutant tumours, using the dTAG system. The key finding is that the tag fusion impairs some aspects of RAF1 function even in the non-induced state, resulting in differences in tumour sensitivity between animals carrying tagged versus wildtype alleles even before protein degradation is induced. This is rationalised by showing that the tag has minimal effects on kinase function but does affect protein-protein interactions, consistent with previous results indicating that the scaffold function of RAF1 is critical for tumourigenesis. Degradation of dTAG-RAF1 has little impact on the progression of tumours that do emerge in this model, indicating that degradation of a subfunctional protein in this case does not phenocopy the effect of genetic deletion.

Overall, the study is well conducted and the conclusions are justified. It builds on a growing body of literature highlighting that the fusion of tags to proteins can impair their function, and that the consequences of tag fusion in vivo can be challenging to predict based on in vitro data. I believe the study is suitable for publication in its current form and have only one suggestion for the discussion.

For the benefit of other researchers hoping to avoid the types of problems described in this manuscript, I would encourage the authors to further develop their discussion of how their learnings might inform the allele design process. They compared C- and N-terminal tag fusion but there is theoretically much more that could be done to minimise the level of perturbation introduced by the tag. It is beyond the scope of the current study to explore this experimentally, and I acknowledge that this point is already touched upon in the current discussion (lines 287 - 290). But specifically, how might the authors incorporate structural insights into future efforts to design a tagged RAF1 protein that retained scaffolding function? Are there strong candidate sites for internal fusion, and how might the authors prioritise them?

We thank the reviewer for the careful evaluation and positive assessment of our study. We also thank the reviewer for this constructive suggestion regarding how structural insights could guide the design of future tagged RAF1 alleles.

Accordingly, we have expanded the Discussion to address this point. We now explain that candidate internal insertion sites could initially be prioritized within predicted solvent-exposed or intrinsically disordered regions, while avoiding the structured RAS-binding and cysteine-rich domains, the kinase domain, and critical regulatory motifs such as the region surrounding S259. In RAF1, the disordered region separating its N-terminal regulatory modules from the kinase domain may therefore provide candidate positions for empirical screening. This strategy is also supported by ongoing unpublished work in our laboratory using a small internal HA tag in RAF1.

Nevertheless, no insertion site can be assumed to be functionally neutral based on structural predictions alone. Candidate alleles should therefore be evaluated at physiological expression levels for protein abundance and localization, kinase activity, relevant protein-protein interactions, and the ability to support RAF1-

dependent biological functions before their incorporation into an *in vivo* degron model. We have incorporated these considerations into the revised Discussion.

Reviewer #2 (Comments to the Authors (Required)):

This study investigates dTAG-mediated RAF1 degradation in a genetically engineered KRAS-driven lung adenocarcinoma model. Although pharmacological degradation of dTAG-RAF1 does not provide an apparent therapeutic benefit and fails to reproduce the tumour regression observed following genetic RAF1 ablation, the study nevertheless provides valuable insights into the design and interpretation of targeted protein-degradation approaches. In particular, the finding that introduction of a degradation tag may itself alter the biological properties of the target protein represents an important methodological consideration with relevance beyond RAF1. Overall, the study is carefully designed, supported by substantial in vivo and cellular experimentation, and the data are generally clearly presented and figures are generally clearly presented. However, several experimental and mechanistic aspects require clarifications to fully support their main conclusions.

The authors appear to argue that the N-terminal FKBP12F36V tag perturbs RAF1 scaffold functions. At the same time, RAF1 is also maintained in a specific conformational state by the HSP90-CDC37 complex acting on the kinase domain. It would therefore be informative to determine whether the N-terminal tag alters this complex and whether changes in chaperone occupancy could indirectly remodel the RAF1 interactome and contribute to the altered scaffold behaviour observed.

We thank the reviewer for raising this mechanistic possibility. Our existing data indicate that the N-terminal tag does not prevent dTAG-RAF1 from associating with the HSP90-CDC37 complex. Both proteins were recovered in both the WT-RAF1 and dTAG-RAF1 interactomes and were not among the interactions depleted in the dTAG-RAF1 condition (Fig 5A). In addition, to address this point, we examined the composition of affinity-purified WT RAF1-HSP90-CDC37 and dTAG-RAF1-HSP90-CDC37 complexes used for the *in vitro* kinase assay. Both complexes were generated under the same experimental conditions and purified through the same C-terminal Strep affinity tag. Coomassie staining showed that HSP90 and CDC37 co-purified with both WT RAF1 and dTAG-RAF1, with no evident qualitative loss of either chaperone in the presence of the N-terminal tag (Reviewer Response Figure 1).

We acknowledge that this qualitative analysis does not constitute a measurement of complex stoichiometry and cannot exclude more subtle effects on the conformational state of RAF1 within the RHC complex. Nevertheless, the data argue against a major disruption of this complex formation as the primary explanation for the altered interaction profile of dTAG-RAF1. We have included the corresponding Coomassie-stained gel below for the reviewer's assessment.

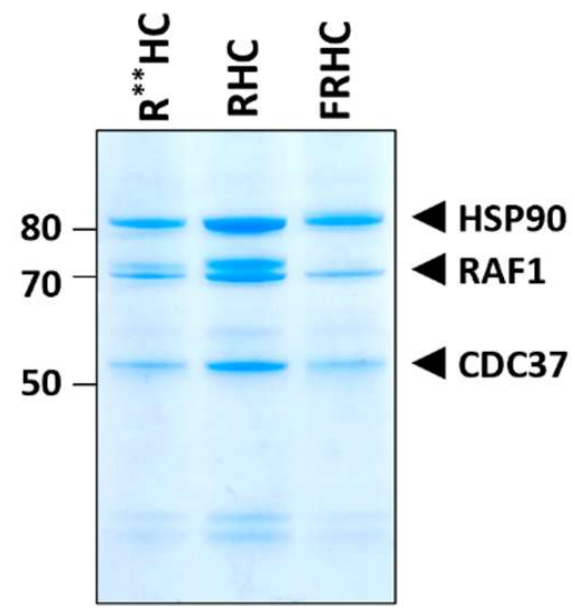

Reviewer Response Figure 1. Composition of affinity-purified RAF1-HSP90-CDC37 (RHC) complexes. Coomassie-stained SDS-PAGE gel showing the indicated RHC complexes purified through the C-terminal Strep affinity tag. The positions of the different proteins are indicated. R**, RAF1$^{Y340E/Y341E}$ constitutively active mutant; R, WT RAF1; FR, dTAG–RAF1; H, HSP90; C, CDC37.

The structural analysis of RAF1 ubiquitination sites appears disconnected from the central mechanistic story, as no experimental evidence links K462/K575 to dTAG-mediated degradation or the altered scaffold function of tagged RAF1. This section therefore adds unnecessary complexity and could be moved to the Extended View or omitted unless its functional relevance is demonstrated.

We agree that our analysis does not establish a functional link between K462/K575 and either dTAG-mediated degradation or altered RAF1 scaffold function. As suggested, this exploratory analysis is already confined to the Extended View section (referred to from now on as Supplementary Figure 2, Fig S2).

The authors state that the Raf1L/L and Raf1−/− data derive from historical cohorts generated in their laboratory. Were these data previously published? If so, the original study should be cited. Authors should explicitly acknowledge here (not only later in the Discussion) that these cohorts were generated and analysed at different times and provide sufficient information to assess their comparability, including genetic background, age/sex distribution, tumour-induction protocol, imaging schedule and experimental endpoints. These historical data should not be presented as directly equivalent to current study control groups unless such comparability can be demonstrated.

We thank the reviewer for this important clarification. The historical *Raf1*$^{L/L}$ and *Raf1*$^{-/-}$ data were previously published in Sanclemente *et al.* (2018). We agree that the basis and limitations of their comparability should be stated at the point where these data are presented, and we have therefore added the original citation and a brief clarification to the Results.

These cohorts are not presented as contemporary control groups or as statistically equivalent to the current dTAG-Raf1 cohorts. They are explicitly identified as historical and are included only to provide context for the previously established response to genetic Raf1 ablation. The figure legend also states that direct comparison is limited by differences in experimental design, treatment modality and cohort size. Statistical analyses were restricted to the matched groups within each study (*Raf1*$^{L/L}$ versus *Raf1*$^{-/-}$ in the historical study and vehicle

versus dTAGV-1m in the current study) and no statistical comparison was performed between historical and current-study cohorts. Full information regarding the genetic background, age and sex of the animals, tumor-induction protocol, imaging schedule and experimental endpoints of the historical cohorts is provided in the cited original publication.

Fig. 1: The authors attribute the altered functionality of dTAG-RAF1 specifically to the N-terminal FKBP12F36V part. However, the dTAG-RAF1 construct also contains an N-terminal 2×HA tag. An appropriate matched control (e.g. 2×HA-RAF1) would be the ideal one to distinguish effects caused by FKBP12F36V from those resulting from N-terminal tagging per se. 1B. To compare the RAF1 levels the samples should have been analysed on the same membrane under identical exposure conditions, with appropriate quantification. Separate blots cannot establish comparable protein abundance reliably.

We thank the reviewer for raising these points. Regarding the composition of the N-terminal tag, we agree that a matched 2xHA-RAF1 allele would be required to distinguish formally between effects caused by the 2xHA sequence and those caused by FKBP12^{F36V}. However, generating and characterizing an additional knock-in mouse model carrying only the 2xHA sequence would be beyond the scope of the present study. Importantly, our conclusions concern the functional consequences of introducing the complete N-terminal cassette used in the dTAG system, rather than either component in isolation. We have clarified the terminology in the manuscript to refer consistently to the complete N-terminal dTAG cassette and to avoid assigning the observed effects exclusively to FKBP12^{F36V}.

Regarding Fig 1B, the protein-abundance comparison was not based on visual comparison of the two representative western blot panels. In each experiment, the RAF1 or dTAG-RAF signal was quantified relative to the corresponding vinculin loading control present on the same membrane. The graph in the lower panel represents the mean ± SEM of these normalized measurements from two independent experiments. We have clarified the normalization procedure, number of independent experiments and origin of the samples in the revised figure legend.

In addition, 4-OHT-induced recombination/RAF1 depletion should be demonstrated alongside the presented blots to verify that RAF1 deletion occurred.

We would like to clarify that the samples shown in Fig 1B were obtained before any 4-OHT-induced recombination or degrader treatment. The unrecombined Raf$^{L/L}$ allele therefore expresses WT RAF1 and was intentionally used to compare its basal abundance with that of dTAG-RAF1 in dTAG-RAF1$^{KI/KI}$ tissues.

1D-E. The choice of the historical Raf1L/L cohort as the comparator requires further justification. Differences in genetic background and experimental conditions between the dTAG and historical RAF1L/L cohorts could otherwise contribute to the striking difference in tumour penetrance/onset.

We thank the reviewer for raising this point. The historical *Raf1*$^{L/L}$ cohort was included because the Kras^{G12V}-driven, Trp53-null LUAD platform has been routinely used in our laboratory across multiple studies and independently

generated compound mouse strains. Across these cohorts, CT-detectable tumors reproducibly emerge approximately 5-6 months after viral induction (Sanclemente *et al.*, 2018; de-la-Puente-Ovejero *et al.*, 2026), providing a well-established temporal benchmark for tumor onset in this oncogenic context.

We acknowledge that the historical *Raf1*$^{L/L}$ and current *dTAG-Raf1*$^{KI/KI}$ cohorts were not generated contemporaneously and that potential effects of genetic background or other cohort-specific variables cannot be formally excluded. The comparison in Fig 1D is therefore descriptive rather than statistical. Nevertheless, tumor penetrance in the *dTAG-Raf1*$^{KI/KI}$ cohort deviated markedly from the kinetics consistently observed across previous cohorts, and increasing the viral dose tenfold did not restore the expected penetrance. We have clarified both the rationale for using the historical cohort and the limitations of this comparison in the revised Results.

Fig. 2. The figure legend should explicitly state whether error bars represent SD or SEM, as these provide substantially different visual representations of variability. Although the Statistics section indicates that data are generally presented as mean {plus minus} SEM, variability differs markedly among panels. But looking at error bars e.g., A versus B, high variations are visible. For these physiological measurements, showing individual data points together with mean {plus minus} SD would provide a more informative representation of biological variability. B-D. The Day 0 baseline data require clarification. The same animals appear to show relatively limited variability in body weight at Day 0, whereas fat mass and BMD show substantially greater and, importantly, markedly different dispersion between the vehicle and dTAGV-1m groups before treatment has begun. Please show the individual animal values, specify whether the error bars represent SD or SEM, and provide the sex distribution of each group. For these longitudinal measurements, connecting individual animals across time would also substantially improve interpretation. E-F. Please clarify the meaning of the different symbols (triangles in E and circles in F).

We thank the reviewer for this helpful suggestion. We have revised Fig 2B-F to display the individual values for each animal. The figure legend now explicitly states the meaning of the error bars and specifies the sex distribution of each experimental group. We have also clarified that Day 0 corresponds to the baseline measurements obtained before treatment initiation. The apparent differences in dispersion at day 0 therefore reflect the biological variability within these small cohorts and can now be directly assessed from the individual data. Finally, we have standardized the symbols across panels B-F and provided the sex distribution.

Fig. 3A-B. Since RAF1 degradation does not reduce MEK1/2-ERK1/2 phosphorylation, the possibility of compensatory signalling through the other RAF isoforms should be addressed. The activation/phosphorylation status of RAF proteins, particularly BRAF and RAF1 where appropriate, should be analysed, especially when talking about these RAFs in the proposed model about compensation.

We thank the reviewer for raising this important point. As shown in Fig 3A, dTAG-RAF1 degradation did not produce consistent changes in total BRAF or ARAF

abundance, while MEK1/2 and ERK1/2 phosphorylation remain preserved. Since MEK1/2 are the direct downstream substrates of RAF kinases, maintenance of phospho-MEK1/2 provides a functional readout demonstrating that the remaining RAF isoforms are sufficient to sustain canonical MAPK signaling following RAF1 degradation. This is consistent with our previous observations following genetic *Raf1* ablation (Sanclemente *et al.* 2018 and 2021).

Of note, the activation state of individual RAF proteins cannot be reliably inferred from western blot analysis of a single phosphorylation site. RAF proteins are regulated through a complex combination of activating and inhibitory phosphorylation events, dimerization, subcellular localization and interactions with regulatory proteins. Therefore, analysis of individual phospho-RAF epitopes could provide an incomplete or potentially misleading representation of their net kinase activity (Riaud et al., 2024 DOI: 10.1038/s41568-023-00650-x).

Fig. 3C-E. Although the numbers of mice and tumours analysed are provided, the presentation is somewhat difficult to follow because the number of tumours varies considerably between analyses. The authors should clearly indicate how many tumours originate from each individual mouse and, where possible, identify tumours belonging to the same animal in the plots. This is particularly important because multiple tumours derived from the same mouse cannot be considered fully independent biological replicates and the hierarchical structure of the data should be appropriately accounted for in the statistical analysis.

We agree that both the number of mice and the number of tumors should be explicitly reported. We have therefore revised the legend to Fig 3C-E to provide both values for each analysis.

This autochthonous multifocal LUAD model generates spatially distinct lesions that can display heterogeneous responses and are therefore longitudinally monitored as individual tumor-response units by CT. This lesion-level representation has been consistently used in previous studies employing the same experimental platform (Sanclemente et al., 2018, 2021; Esteban-Burgos et al., 2020; Salmón et al., 2023; de-la-Puente-Ovejero et al., 2026).

Rather than introducing additional mouse-specific symbols that would increase the visual complexity of the panels, we have retained the established lesion-level representation while reporting both the number of animals and the number of tumors contributing to every analysis. Fig 3C presents the longitudinal trajectory of each tumor, and the longitudinal analysis in Fig 3D was performed using a linear mixed-effects model. We believe that these clarifications provide the necessary transparency regarding the structure and composition of the datasets.

Fig. 4A. Appropriate controls are required to establish that the observed PARP and caspase-3 cleavage results specifically from Raf1 deletion rather than prolonged 4-OHT exposure. In addition, the strongest apoptotic phenotype following genetic Raf1 deletion is observed at 96 h, whereas dTAGV-1m-treated cells are analysed only up to 72 h. A matched 96-h dTAGV-1m time point is required before concluding that acute RAF1 degradation fails to reproduce the apoptotic phenotype caused by genetic deletion. Some literature data indicate that 4-OHT itself may induce apoptotic/cell-death

responses (PMID: 23722551). Given the prolonged treatment (up to 96 h), such effects cannot be excluded, particularly since the 4-OHT concentration used is not clearly provided in the Methods. An appropriate 4-OHT-treated control in which Raf1 cannot be recombined should therefore be included to distinguish potential treatment-related effects from apoptosis specifically caused by RAF1 depletion. 4B. This experiment was performed only once and therefore cannot support a quantitative conclusion regarding apoptosis.

We thank the reviewer for raising these points. The genetic *Raf1* ablation time course shown in Fig 4A was included as a previously established positive reference rather than as a new demonstration of the specificity of 4-OHT-induced apoptosis. This conditional system and its apoptotic phenotype were characterized previously by Sanclemente *et al.* (2018) using three independent tumor-derived cell lines from the same *Kras*^{G12V}/*Trp53*^{null} LUAD model employed here. In that study, progressive loss of RAF1 was consistently accompanied by the appearance of cleaved caspase-3 in cultured tumor cells, and apoptosis following *Raf1* ablation was also demonstrated *in vivo*. The present experiment reproduces the same temporal association between RAF1 loss and caspase-3 cleavage.

We acknowledge that 4-OHT can exert biological effects in certain cellular contexts. However, the study cited by the reviewer examined a different tumor type and described autophagic cell death associated with KRAS degradation. In our experiment, conditional *Raf1*^{L} LUAD cells were treated with 600 nM 4-OHT using the same established CreERT2-dependent ablation protocol and time course previously validated in this model.

The 4-OHT concentration, which was insufficiently specified in the original manuscript, is now explicitly reported as 600 nM in the Materials and Methods and in the figure legend.

Regarding the different time courses, the sampling points were selected according to the kinetics of RAF1 elimination rather than simply matching the duration of compound exposure. Following genetic recombination, RAF1 depletion is progressive, and cleaved caspase-3 first becomes detectable concomitantly with substantial RAF1 loss at approximately 48 h, becoming more pronounced at later time points. By contrast, dTAG^{V}-1m induces complete dTAG-RAF1 degradation within 2 h. Thus, by the 48-h endpoint, dTAG^{V}-1m-treated cells had remained essentially depleted of RAF1 for approximately 46 h, including the period at which apoptosis first becomes detectable in the genetic model, without detectable accumulation of cleaved caspase-3. We therefore consider that extending dTAG^{V}-1m treatment to 96 h is not required to assess the acute apoptotic response associated with RAF1 elimination. This interpretation is further supported by the prolonged *in vivo* degradation experiments, in which sustained dTAG-RAF1 loss did not induce tumor regression.

We have revised the figure legend to clarify the rationale for the different time courses.

Finally, we agree that Fig. 4B, which was performed once, should not be interpreted quantitatively. This panel was intended as a qualitative, exploratory observation and was not subjected to densitometric quantification or statistical analysis.

The Annexin-V/PI analysis should be repeated in at least three independent biological experiments, with quantification and appropriate statistical analysis. 4C. The flow-cytometry gating strategy is not provided. Representative Annexin V/PI plots together with the complete gating strategy, including cell/singlet selection, compensation and the controls used to establish the Annexin V/PI gates, should be included. This is particularly important because these experiments are used to support the central conclusion that dTAG-mediated RAF1 degradation does not induce apoptosis.

We agree that repeating the Annexin V/PI analysis in independent biological experiments would be required to draw a quantitative conclusion and perform meaningful statistical analysis. The experiment shown in Fig 4C was performed once and was intended as an exploratory, orthogonal assessment of apoptosis rather than as independent quantitative evidence. We have therefore clarified in the figure legend that this panel represents a single experiment, has not been subjected to statistical analysis and should be interpreted qualitatively.

For transparency, we provide the complete available staining controls and gating plots as a Response Figure for the reviewer's assessment. Cellular events were initially selected using an FSC-H/SSC-H gate to exclude debris. Compensation between the Annexin V-FITC and PI channels was established using Annexin V-only and PI-only controls, and an unstained control was used to position the quadrant gates. A separate singlet-discrimination gate was not applied in this exploratory experiment. Annexin V^{-}/PI^{-} cells were classified as viable, Annexin V^{+}/PI^{-} cells as early apoptotic, and PI-positive populations as late apoptotic or dead. Etoposide-treated samples were included as a positive apoptosis control.

"PDF for reviewer". Staining controls and gating plots for the Annexin V/PI experiment shown in Fig 4C. Page 1 shows the unstained, PI-only and Annexin V-only controls used to establish compensation and position the quadrant gates. Page 2 shows Raf1^L (upper panels) and Raf1-deleted cells (lower panels) treated with DMSO (left) or etoposide (right). Page 3 shows dTAG-Raf1^KI cells treated with DMSO (left) or etoposide (right) in the absence (upper panels) or presence of dTAGV-1m (lower panels). Cellular events were selected using an FSC-H/SSC-H gate to exclude debris. A separate singlet-discrimination gate was not applied. The experiment was performed once.

Fig. 5. The interpretation of the proteomic data in relation to apoptosis is unclear. The authors propose that the N-terminal tag compromises RAF1-mediated anti-apoptotic scaffold functions. However, several proteins annotated as negative regulators of apoptosis appear more abundant in the dTAG-RAF1 interactome. How do the authors reconcile this enrichment with their proposed model? The directionality of the individual proteins contributing to the pathway enrichment should be presented and discussed rather than interpreting pathway enrichment alone as evidence for impaired anti-apoptotic RAF1 function.

We agree that pathway enrichment alone does not provide information about the direction of individual interaction changes or establish their net functional effect on apoptosis. Although Fig 5C displays the directionality of the individual proteins through their $\log_2$ fold change, this was not sufficiently explained in the original text. We have modified the figure legend to clarify this.

Inspection of the individual proteins reveals a bidirectional remodeling of the apoptosis-related RAF1 interactome. Several proteins annotated as negative regulators of cell death were more abundant in the dTAG-RAF1 interactome. Conversely, other negative regulators showed reduced association. It is important to keep in mind that functional annotations describe the reported role of each protein in cell-death regulation and do not by themselves establish the functional consequence of its interaction with RAF1. Notably, the interaction with ASK1, a pro-apoptotic kinase whose activity is inhibited through its association with RAF1, was also reduced in the dTAG-RAF1 condition.

An increased association with proteins annotated as negative regulators of apoptosis does not necessarily imply enhanced anti-apoptotic dTAG-RAF1 activity. Such changes could reflect compensatory recruitment, altered sequestration or the formation of non-productive protein complexes, possibilities that cannot be distinguished by interaction proteomics alone. The proteomic data demonstrate altered scaffold organization and are interpreted together with the functional and *in vivo* observations.

LUAD cell lines: The generation and characterization of the tumour-derived LUAD cell lines should be described in substantially greater detail. How many independent cell lines were established from individual tumours/mice, how were they authenticated/characterized, and how many independent lines were used for the experiments? EV3A-B appears to show only one representative line. If the conclusions in Fig. 4 are based predominantly on a single tumour-derived cell line, validation of the key findings in independently derived lines would substantially strengthen confidence in the conclusions.

We thank the reviewer for pointing out that the origin and use of the tumor-derived LUAD cell lines were insufficiently described. We have substantially expanded the Materials and Methods to clarify their origin, establishment and characterization. Four independent cell lines were established from each LUAD model, each originating from a separate tumor in a different mouse. However, not all established lines were used in every experiment.

Fig EV3A-B (Fig S3A-B in the revised version) shows one representative *dTAG-Raf1*^{KI} line. In Fig 4A, dTAG-RAF1 degradation and the absence of a detectable apoptotic response were examined in two independently derived *dTAG-Raf1*^{KI} lines, alongside one independently derived conditional *Raf1*^{L} line undergoing genetic ablation. The mechanistic experiments shown in Fig 4B-C were performed using one independently derived cell line from each genotype.

The relevant RAF1 status was verified by immunoblotting, including confirmation of dTAG-RAF1 degradation following dTAG^{V}-1m treatment and RAF1 elimination following 4-OHT induced recombination.

We agree that extending all mechanistic analyses to additional independently derived lines would provide further validation. Nevertheless, the conclusions are supported by several complementary levels of evidence, including the *in vivo* tumor phenotype, efficient dTAG-RAF1 degradation in tumor samples, the absence of an apoptotic response, and the biochemical and interactome analyses. We have revised the text to avoid generalizing the cell-based observations beyond the models analyzed.

Cell models and reproducibility. The manuscript does not clearly state whether lentivirally generated cell lines represent polyclonal populations or individually selected clones, nor how many independently generated cell populations/clones were analysed. Since lentiviral transduction results in random genomic integration and potentially variable transgene copy number/expression, conclusions concerning functional differences between RAF1 constructs should not rely on a single independently generated line/clone. The authors should clarify how the cell lines were generated, report the number of independent clones/populations tested, and ideally demonstrate the key phenotypes in independently generated lines.

We thank the reviewer for raising this point. Lentiviral transduction was used only for the RAFless rescue assay shown in Figs EV1 and EV3C (S1 and S3C in the revised version). Following transduction and antibiotic selection, cells were maintained as pooled polyclonal populations, and no individual clones were isolated. Each assay was repeated at least twice, with each replicate involving an independent lentiviral transduction and antibiotic selection.

Importantly, the tumor-derived LUAD cell lines were not generated by lentiviral transduction. These lines were established directly from tumors arising in the corresponding genetically engineered mouse models and express RAF1 or dTAG-RAF1 from the endogenous *Raf1* locus. Thus, the principal *in vivo* and tumor-derived cell findings are not subject to clonal effects resulting from random lentiviral integration.

We have revised the Material and Methods and the corresponding figure legends to clarify these points.

Fig. 1C. It is unclear why Raf1 expression is presented as -ΔΔCt. Please clarify also the calculation in methods part.

We thank the reviewer for pointing out that the presentation and calculation of the qRT-PCR data were insufficiently explained. *Raf1* expression was calculated using the comparative threshold cycle method. Briefly, *Raf1* Ct values were normalized to *Rn18s* to obtain ΔCT values, and the mean of the *Raf1*$^{L/L}$ control group was used as the reference to calculate ΔΔCt. Relative expression can therefore be expressed as $2^{-\Delta\Delta CT}$ or, as shown in Fig 1C, as -ΔΔCT, which corresponds to $\log_2$-transformed relative expression and centres the control group around zero. We have now added a dedicated qRT-PCR subsection to the Materials and Methods and clarified the calculation and data representation in the figure legend.

Fig. EV3E. Cells and nuclei in the upper panel appear considerably smaller than those in the two lower panels. Please clarify whether all images were acquired and displayed

at the same magnification and whether the indicated scale bar applies equally to all panels. If the scale is indeed identical, is the apparent difference in cell/nuclear size a reproducible phenotype associated with the different RAF1 conditions?

We thank the reviewer for this observation. All images in Fig EV3E (Fig S3E in the revised version) were acquired and displayed at the same magnification, and the indicated 20 µm scale bar applies equally to all panels. The apparent difference in cell and nuclear size was not consistently observed across fields and is not considered a reproducible phenotype associated with the different RAF1 conditions.

The study examines one scaffold protein, one N-terminal tag configuration and one degrader; the conclusions therefore cannot yet be broadly generalized to degron-based approaches. I would encourage the authors to soften the language accordingly in relevant parts of the manuscript.

We agree that our study examines one particular case and therefore does not support broad generalization to all degron-based approaches. We have revised the relevant sections of the manuscript to soften statements implying general applicability. We also explicitly acknowledge this limitation in the Discussion.

Row 158: "... suggesting that RAF1 is dispensable for normal tissue homeostasis". I consider that should not be generalised, should be used something like "under the conditions examined".

We thank the reviewer for this comment. Although the lack of detectable toxicity is consistent with previous genetic studies from our group, we agree that this conclusion should be restricted to the experimental conditions examined. We have revised the sentence accordingly.

The Discussion reads in several places as an extended summary of the Results. It would benefit from being streamlined and refocused on interpretation, limitations, alternative explanations and the broader implications of the findings.

We have revised and streamlined the Discussion to reduce repetition of the Results and place greater emphasis on the interpretation, limitations, and broader implications of our findings.

Methods/Western Blotting: Separate antibodies against MEK1, MEK2, ERK1 and ERK2 are listed in Materials/Methods, whereas the figures label signals as MEK1/2 and ERK1/2. Please clarify whether antibodies were combined for detection or whether a single isoform-specific antibody is being used to represent MEK1/2 or ERK1/2.

The isoform-specific antibodies against total MEK1 and MEK2 were combined for detection of total MEK1/2, and the antibodies against total ERK1 and ERK2 were similarly combined for detection of total ERK1/2. By contrast, phosphorylated MEK1/2 and ERK1/2 were detected using single antibodies that recognize both phosphorylated isoforms. We have now clarified this information in the Western blotting Methods section.

IP/MS analysis: The Methods state that either anti-RAF1 or anti-HA magnetic beads were used for immunoprecipitation. Please specify which antibody was used for each

condition. If WT RAF1 and dTAG-RAF1 interactomes were generated using different affinity reagents, the rationale and appropriate controls should be provided, as this may influence recovery of RAF1 and associated proteins.

We thank the reviewer for requesting this clarification. The primary WT RAF1 versus dTAG-RAF1 interactome comparison was performed using the same anti-RAF1 antibody in both conditions. Anti-HA beads were used only as an orthogonal affinity control, together with the corresponding HA-negative control. We have substantially expanded the Materials and Methods to specify the antibody used in each condition and the rationale for the associated controls.

Reviewer #3 (Comments to the Authors (Required)):

The work by de-la-Puente-Ovejero et al. describes the use of a chemogenetic approach to delete Raf1 in mice and explore its consequences and applicability as a therapeutic avenue in Ras-driven tumorigenesis. This follows up on a growing body of data originating from the authors´ and other laboratories pointing to Raf1 kinase inhibition as a promising therapeutic strategy for Ras driven cancers. Specifically, the authors generated a dTAG-RAF1 genetic mouse model and combined this with a previously well-established inducible KRasG12V system to induce lung adenocarcinoma (LUAD). Using this approach they show that Raf1 can be effectively removed chemogenetically without inducing overt toxicity in the animals, consistent with the results from genetic knockdown strategies applied previously by the same group and others. The chemogenetic deletion of Raf1 protein did however not significantly affect tumour growth, which was less pronounced in the dTAG-RAF1 animals in the first place. The authors conclude that the N-terminal appendage of the dTAG compromises protein-protein interactions that are essential to mediate the non-catalytic Raf1 function in curbing apoptosis. The authors substantiate this notion by a number of additional experiments including a proteomic comparative analysis of the RAF1 versus dTAG-RAF1 Interactome.

The study is well-designed and technically sophisticated. All of the experiments, as well as the interpretation and analysis of the data, are well-founded, sound and support the conclusions drawn. The finding that the chemogenetic deletion of Raf 1 is not toxic represents an important observation, given the promising prospects for RAF1 as a target molecule in Ras-dependent tumors. However, in my opinion, the central finding-that the dTAG-RAF1 fusion construct lacks a large portion of its anti-apoptotic functions due to the loss of scaffold integrity-is somewhat overrated in terms of novelty. This does however not negate the validity, soundness and relevance of the findings as the authors make a good job in rationalizing all findings around the mentioned scaffold integrity framework. I like also the final summary figure 6, which I believe is very helpful for the reader.
Apart from that, I have no major issues or comments, as I believe that all the experimental data presented are fundamentally sound, justify the conclusions and will be of interest to readers in the fields of Ras/Raf and cancer research. At most, I would recommend that the authors tone down the novelty of the loss of scaffold integrity somewhat and place it in the correct context, as this essentially reflects the well-known fact that C- or N-terminal tags are likely to influence the function of the target protein - a fact that has long been recognised by researchers in the field of signal transduction.

We thank the reviewer for the thoughtful and supportive assessment of the study and for recognizing the relevance of the findings to the RAS/RAF and cancer research fields. We agree that the general ability of N- or C-terminal fusion tags to perturb protein function is well established, and we did not intend to present this principle itself as a novel finding. We have therefore toned down the corresponding novelty claims and placed our results in the appropriate context. We have also restricted our conclusions to the preclinical models studied and discussed the potential implications for previous studies employing tagged RAF constructs.

The specific changes introduced in response to these points are detailed below.

In addition, I have identified a number of **minor points** which I recommend the authors correct in a revised version of the manuscript:

• Since the study describes the effects of a Raf1 fusion protein on the integrity of the Raf1 scaffold, the conclusions primarily pertain to preclinical contexts. I would suggest limiting the focus and conclusions mainly to experimental models. The authors may consider also making this point clear in the title of the manuscript, for example, by editing such as this or something similar: RAF1 scaffold integrity shapes chemogenetic degradation outcomes in preclinical models of KRAS-driven lung cancer

We agree that the conclusions of this study primarily concern the experimental models examined here. We have therefore revised the title to make the scope of the work explicit.

• The results presented here also have numerous implications for earlier studies that used tagged Raf1. Many researchers, including this reviewer, have used N-terminally tagged Raf constructs in their studies, and some of these studies may need to be revisited. It might be helpful to briefly mention this fact.

We agree that our findings may have implications for the interpretation of earlier studies employing N-terminally tagged RAF constructs, so we have now briefly acknowledged this point in the Discussion, while clarifying that our results don't imply that all N-terminal tags necessarily perturb RAF function.

• Immunoprecipitation and LC-MS/MS analysis: In my view, this part requires more experimental detail. The experiments were done in "in LUAD-derived cell lines" according to the main text. What cells exactly were used? Could the authors provide more detail in the experimental section? Why were different antibodies (HA vs. Raf1) used to pulldown WT Raf1 or dTAG-Raf1? Using different Abs for IP can affect the associated proteome differently, much along the argument of scaffold integrity made by the authors here in the paper. Also, was native WT RAF1 present in the dTAG-RAF1 cells used for the pulldown? In that case there would be a situation of competition for interactors between native and heterologous dTAG-RAF1. Would it not make sense to use the Rafless cells +/- dTAG-RAF1 allowing to pulldown WT Raf and dTAG-RAF1 with the same α-Raf1 antibody to exclude all these confounders? Please specify and comment. Also I would suggest to provide more detail on the data analysis, e.g. were the peptide lists normalized internally? E.g. to the RAF1-bait peptide frequency?

We thank the reviewer for these comments. We have substantially expanded the Materials and Methods to specify the LUAD-derived cell models, immunoprecipitation conditions, controls and data-analysis workflow.

Importantly, the primary WT RAF1 versus dTAG-RAF1 comparison was performed using the same anti-RAF1 antibody. The anti-HA pulldowns were included as an orthogonal control to identify affinity-reagent-dependent protein recovery. Moreover, the *dTAG-Raf1*$^{KI/KI}$ cells express dTAG-RAF1 from the endogenous homozygous knock-in locus and do not contain native WT RAF1; therefore, there is no competition between WT and dTAG-RAF1.

We have also clarified that protein-group abundance was quantified using MaxLFQ rather than raw peptide counts. Differential abundance was analyzed using the resulting MaxLFQ values, whereas fold changes were expressed relative to RAF1 bait recovery for visualization in Fig 5A. Full details are now provided in the revised Material and Methods.

• L77: “Second, the biological outcome of degradation depends not only on efficient target elimination, but also on whether loss of the protein reproduces the relevant functional consequences of genetic ablation." This sentence is ambiguous and its exact meaning is not clear to me.

We thank the reviewer for pointing out the ambiguity of this sentence. Our intention was to indicate that efficient elimination of a protein does not necessarily reproduce the phenotype caused by genetic ablation of the corresponding gene. We have revised the sentence to convey this distinction more clearly.

• L86: "Indeed, emerging evidence suggests that proteins carrying chemogenetic tags may exhibit altered function even prior to degradation" This sentence illustrates my previous point: The fact that an attached tag can influence the function of a bait protein-whether it is a scaffold protein or another type of protein-is not a new finding but has been known for many years. I suggest toning down these claims a little.

We fully agree with the reviewer that the ability of N- or C-terminal fusion tags to perturb protein function is a long-established principle. Our intention was instead to emphasize the specific consequences of this issue in the experimental system studied here. We have revised the Introduction to tone down the corresponding novelty claim.

• Fig1A: The mouse model Is well illustrated but I would suggest to include further information e.g. in the figure legend, making clear to readers not familiar with Raf1 genetics that the ATG start codon resides in exon 2.

We thank the reviewer for this suggestion. We have now clarified in the Fig 1A legend that the endogenous *Raf1* translation initiation codon is located in exon 2.

• L141: " ...in parallel, a second compound, IGP002 (a PROTAC analogue with improved solubility and metabolic..." Can the authors please include a reference for IGP002?

The reference describing IGP002 was already included in the manuscript but was inadvertently placed at a subsequent mention of the compound. We have now moved the citation to its first mention in the Results section.

• The Caption in Fig 3E is ambiguous and should be made more explicit to better describe the four groups/columns.

We agree with the reviewer and have revised the Fig 3E legend to identify explicitly the four groups represented, their order and color coding, the intervention applied in each model, and the calculation and interpretation of the fold change in tumor volume. We have also clarified that n refers to individual tumors and that statistical comparisons were performed only within the historical and contemporaneous datasets, respectively.

• In vitro kinase assays: Fig3D should include a load of rMEK by Coomassie or similar.

We agree with the reviewer that direct visualization of total rMEK by Coomassie staining or immunoblotting would strengthen this experiment. Unfortunately, this control was not acquired when the assay was performed, and we are unable to generate it retrospectively. However, all kinase reactions were assembled in parallel using identical amounts of recombinant MEK1 dispensed from the same preparation. We have clarified this aspect in the Methods and figure legend.

In addition, because equal substrate loading cannot be directly documented in the blot, we have revised the Results to describe this experiment as a qualitative assessment and have avoided interpreting it as evidence of quantitatively equivalent kinase activity.

• The authors describe and document a lack of toxicity of Raf1 depletion, consistent with previous genetic Raf1 ablation data. This suggests that the apoptosis-regulatory role of Raf1 becomes only relevant in the context of oncogenic Ras signalling in tumorigenesis. Analogous selective effector engagement by oncogenic versus wt Ras has been suggested previously e.g. for PI3K (PMID: 30590030; PMID: 17540175; PMID: 30560710). All these data suggest that oncogenic Ras not just simply overactivates its effectors but rather it appears that it re-wires and re-purposes downstream effectors for oncogenesis. This is an intriguing implication which the authors might like to shortly comment on in the discussion part.

We thank the reviewer for this insightful suggestion. We agree that the contrasting consequences of RAF1 loss in normal tissues and KRAS-driven tumors raise this intriguing possibility that oncogenic KRAS rewires effector usage. We have briefly discussed this possibility and the analogous context-dependent engagement of PI3K by oncogenic versus WT RAS in the revised Discussion.

• Have the Rafless MEFs been published? Please specify. If not, please provide more background in the experimental part.

These RAFless MEFs are long-established cell lines generated in the Barbacid laboratory and have been used in several previous studies, although their original generation was not formally described. We have now clarified this point in the Methods section, explained their generation in more detail, and included references to previous studies in which these cells were used.

• Some information about the antibodies used for Western Blotting is confusing; e.g. The western image panels indicate pMEK1 while in the methods description a pMEK1/2 Ab is described. Please check and specify.

We have reviewed and standardized the MEK nomenclature throughout the figures and have clarified in the Methods which antibodies were used for total and phosphorylated MEK detection. Signals from cellular or tumor lysates are now consistently labelled as pMEK1/2. In the *in vitro* kinase assay, recombinant MEK1 was the only substrate present, and the corresponding signal is therefore identified as pMEK1.

• Please indicate which cells were used in Fig 4. Were these MEFs?

The experiments shown in Fig 4 were performed using primary mouse LUAD-derived cell lines, not MEFs. We have now specified the identity and genotype of these cells in the Methods section and in the Fig 4 legend.

September 2, 2026

RE: Life Science Alliance Manuscript #LSA-2026-03839-TR

Dr. Sara García-Alonso
Spanish National Cancer Research Centre
Tumor Biology Programme
Melchor Fernández Almagro 3
Madrid, Madrid 28029
Spain

Dear Dr. García-Alonso,

Thank you for submitting your Research Article entitled "RAF1 scaffold integrity shapes chemogenetic degradation outcomes in KRAS-driven lung cancer models". It is a pleasure to let you know that your manuscript is now accepted for publication in Life Science Alliance. Congratulations on this interesting work.

The final published version of your manuscript will be deposited by us to PubMed Central upon publication.

Your manuscript will now progress through copyediting and proofing. At the proofing stage, we strongly encourage you to complete the section on description of 'RNA extraction and qRT-PCR' (lines 510-526) by including information on primers used for qRT-PCR.

It is journal policy that authors provide original data upon request.

Your article will publish open access upon publication under a CC-BY license.

Reviews, decision letters, and point-by-point responses associated with peer-review at Life Science Alliance will be published, alongside the manuscript. If you do want to opt out of having the reviewer reports and your point-by-point responses displayed, please let us know immediately.

IMPORTANT: If you will be unreachable at any time, please provide us with the email address of an alternate author. Failure to respond to routine queries may lead to unavoidable delays in publication.

Scheduling details will be available from our production department. You will receive proofs shortly before the publication date. Only essential corrections can be made at the proof stage so if there are any minor final changes you wish to make to the manuscript, please let the journal office know now.

DISTRIBUTION OF MATERIALS:
Authors are required to distribute freely any materials used in experiments published in Life Science Alliance. Authors are encouraged to deposit materials used in their studies to the appropriate repositories for distribution to researchers.

You can contact the journal office with any questions, contact@life-science-alliance.org

Again, congratulations on a very nice paper. I hope you found the review process to be constructive and are pleased with how the manuscript was handled editorially. We look forward to future exciting submissions from your lab.

Sincerely,

Sarita Hebbar, PhD
Scientific Editor
Life Science Alliance
http://www.lsajournal.org
